# Changes in the Plasticity of HIV-1 Nef RNA during the Evolution of the North American Epidemic

**DOI:** 10.1371/journal.pone.0163688

**Published:** 2016-09-29

**Authors:** Amirhossein Manzourolajdad, Mileidy Gonzalez, John L. Spouge

**Affiliations:** National Center for Biotechnology Information, National Library of Medicine, National Institutes of Health, Bethesda, Maryland, United States of America; University of South Carolina School of Medicine, UNITED STATES

## Abstract

Because of a high mutation rate, HIV exists as a viral swarm of many sequence variants evolving under various selective pressures from the human immune system. Although the Nef gene codes for the most immunogenic of HIV accessory proteins, which alone makes it of great interest to HIV research, it also encodes an RNA structure, whose contribution to HIV virulence has been largely unexplored. Nef RNA helps HIV escape RNA interference (RNAi) through nucleotide changes and alternative folding. This study examines Historic and Modern Datasets of patient HIV-1 Nef sequences during the evolution of the North American epidemic for local changes in RNA plasticity. By definition, RNA plasticity refers to an RNA molecule’s ability to take alternative folds (i.e., alternative conformations). Our most important finding is that an evolutionarily conserved region of the HIV-1 Nef gene, which we denote by R2, recently underwent a statistically significant increase in its RNA plasticity. Thus, our results indicate that Modern Nef R2 typically accommodates an alternative fold more readily than Historic Nef R2. Moreover, the increase in RNA plasticity resides mostly in synonymous nucleotide changes, which cannot be a response to selective pressures on the Nef protein. R2 may therefore be of interest in the development of antiviral RNAi therapies.

## Introduction

The HIV-1 Nef gene has a single exon of about 620 nucleotides, which partially overlaps a 3’-long terminal repeat. It encodes an immunogenic accessory protein with multiple activities during HIV infection. Although Nef is not essential for viral replication *in vitro*, it is an important virulence factor *in vivo* [1, 2], and it is highly expressed from the early stages of infection [3]. Recent findings have elucidated the poorly understood and complex mechanisms by which Nef enhances viral infectivity [4–6]. Among other mechanisms, it downregulates CD4 cell surface expression and major histocompatibility complex I antigens, and it also modulates T-cell signaling pathways [7]. Although most studies focus on the Nef protein, the Nef gene also encodes an RNA structure, whose contribution to HIV virulence is largely unexplored. Experimental evidence shows that from the early stages of viral replication, Nef sequences appear within HIV mRNAs [8] that may facilitate viral rebound during antiretroviral therapy [9]. Based on computational approaches [10], Peleg et al. [11] noted that Nef RNA contains extensive information beyond the information required merely to encode the Nef protein, and they proposed that the information corresponds to conserved RNA structures.

The reverse transcriptase in HIV lacks double-stranded proofreading, so its high mutation rate generates viral variants, some of which eventually escape selective pressures from the host immune system. The viral variability includes RNA motifs corresponding to “local” RNA secondary structures [12], which are in turn informative of 3D conformations [13]. Viral RNA conformations may in fact be evolving at a much faster rate than the underlying sequence itself [14]. Regardless of evolutionary rates, the prediction and identification of RNA motifs help to construct and to explore important biological hypotheses about the viral life cycle [15–18].

The inherent flexibility of RNA can yield alternative conformations (“folds”) with distinct biological functions. Riboswitches typically have two alternative folds, e.g., for regulating bacterial genes [19–21]. Although viral RNAs are much shorter than bacterial RNAs, with correspondingly less sequence to encode information, their conformational changes can still indicate transitions between competing processes, or even RNA-protein interactions such as RNA processing, among other possibilities [22]. RNA’s ability to assume different low-energy conformations, its “plasticity,” can also be indicative of an ability to evolve [23], since only a few sequence mutations are then required to trigger dramatic conformational rearrangements. HIV-1 RNA may in fact be particularly flexible [24], and several alternative folds may be critical in the HIV-1 life cycle. As examples, the HIV-1 untranslated leader RNA influences RNA dimerization [25, 26]; nascent transcripts of TAR RNA influence transcription and gene expression [27]; and the Rev Response Element modulates nucleocytoplasmic export to control HIV replication rates [28, 29].

RNA interference (RNAi) is a cellular process expressing RNA molecules to inhibit gene expression, typically by accelerating RNA degradation. As such, RNAi provides an evolutionarily conserved defense against viruses [30, 31]. *In vitro*, Nef RNA can evade RNAi with alternative folds [32–34]. *In vivo*, HIV-1 RNA is under selective pressure to evade host-induced RNAi [35] to avoid the downregulated viral replication occurring when certain cellular and Nef RNAs interact [36, 37]. HIV also encodes RNAs, including a Nef-derived miRNA, miR-N367, to regulate its own transcription [38, 39]. Although induced RNAi can inhibit viral replication [40, 41], HIV is resistant to many RNAi-based antiviral therapies [42]. These considerations motivate the present investigation of the evolution of Nef RNA structures and their ability to evade the host immune system via alternative folds.

This article measures RNA plasticity (i.e., the ability to take alternative folds) primarily with a computed quantity “Capacity for Alternative Folds” (CAF). CAF can contribute insights into RNA kinetics, because it incorporates more than just the minimum free energy structure into its measurement of plasticity. To relate changes in RNA plasticity to the evolution of the HIV epidemic, we collected HIV-1 sequences from the Los Alamos Database [43] and from a study on the North American HIV-1 Epidemic [44]. Based on sampling year, we partitioned each of the two datasets into Historic and Modern subsets. If the human immune system places detectable selective pressure on viral plasticity, each Modern dataset should be statistically different from the corresponding Historic dataset. Because the local RNA dynamics observed *in vitro* correspond to local changes to the RNA secondary structure [34], and because local predictions are generally more dependable than global predictions, our study primarily computed local secondary structures from subsequences, rather than global secondary structures from the full-length Nef sequence. Because small changes in a sequence can propagate dramatically to surrounding RNA structures [45], we examined overlapping subsequences.

Many computational approaches to the *ab initio* prediction of alternative folds from RNA sequence exist [46–52]. Although these approaches do not have established success in discriminating riboswitches from genomic background, they have proved useful for investigating the structural features that accompany alternative folding. Ensemble diversity, based on sampling the energy landscape of RNA secondary structures, e.g., can characterize alternative structures in riboswitches [53–55]. Given an RNA sequence, ensemble diversity provides a competitive method for investigating thermodynamically stable alternative folds, and in addition, it has the merits of simplicity and generality. CAF, our measure of RNA plasticity, in fact partially characterizes the diversity in an ensemble of RNA conformations. Our other measures also quantify evolutionary changes in the full-length Nef RNA secondary structure, and in tertiary structural motifs such as pseudoknots and G-quadruplexes (G4).

## Materials and Methods

Ethics statement: The sequences in the Test Datasets were obtained from subjects enrolled under REB-approved protocols who gave written, informed consent in the original studies collecting the specimens. The Institutional Review Boards at Providence Health Care/University of British Columbia and Simon Fraser University granted ethical approval for the study that provided the sequences in the Test Datasets [44]. The Office of Human Subjects Research Protections at NIH determined the present study was excluded from IRB review per 45 CFR 46 and NIH policy.

HIV-1 Nef datasets: Historic and Modern Test Datasets: Cotton et al. [44] classified their cohorts into two datasets according to specimen collection date: a Historic Dataset (1979–1989) and a Modern Dataset (2000–2011). We used the same classification and coordinates (1–621) as their HXB2-alignments. Cotton et al. [44] collected sequences from untreated patients from various locations in North America and verified that the sequences belonged to HIV-1 subtype B. We discarded sequences whose alignments covered < 41% of the HXB2 Nef gene, resulting in Historic and Modern Datasets of 335 sequences each. Table 1 displays additional details about the datasets.

**Table 1:** Features of the Test and Training Datasets of Nef sequences.

Dataset	Name	No. of patients (= Nef sequences)	Collection date	Sampling region	Reference
Test	Historic	335	1979–1989	North America	Cotton et al. [44]
	Modern	335	2000–2011		
Training	Historic	9	1979–1989	Worldwide	Los Alamos Database as of Feb 12, 2015
	Modern	125	2000+		

All Nef sequences are of HIV-1 subtype B and have >95% coverage. No dataset shares a sequence with any other. Sequences of Historic and Modern Datasets are derived from drug-naïve patients. Historic and Modern Datasets for each category do not share a common patient.

Historic and Modern Training Datasets: For the Training Datasets, we used the intra-patient Nef sequence data available in Los Alamos HIV Database as of Feb 12, 2015 [43]. We selected drug naïve patients infected with HIV-1 Subtype B worldwide (a total of 66 studies and 179 patients), with > 95% Nef coverage, from the latest available sampling point (Fiebig stages 4–6). Our statistical tests require independent sampling, so each patient contributed only one sequence. If more than one sequence satisfied our criteria, we selected a single sequence at random. To parallel protocols for the Test Dataset, we split the Training Data into Historic (1979–1989) and Modern (2000+) Training Datasets. No patient contributed to both the Historic and Modern Training Datasets. The Historic Training Dataset contained 9 Nef sequences; the Modern Training Dataset, 125 Nef sequences. HIVAlign [43] aligned the Training Dataset sequences to the HXB2 reference sequence (Genbank accession: K03455). In every procedure described below, we optimized any adjustable parameters with the Training Datasets. Some optimizations were informal (by eye), whereas others had a formal figure of merit. In either case, the corresponding procedure with the Test Dataset always fixed its adjustable parameters and used the values optimized on the Training Dataset. For example, the Modern Training Dataset originally had 136 sequences but we reduced it to 125 to permit a useful but informal optimization from the resulting alignment. The Training and Test Datasets had no sequences in common.

Measuring distance: Hamming distances quantified the dissimilarity between aligned pairs of sequences by the number of differing nucleotides (*d*_*nt*_) or amino acids (*d*_*aa*_). Dissimilarity between two RNA secondary structures can be measured by the number of differing base pairs (*d*_*bp*_) or the tree-editing distance (*d*_*te*_) [56], the minimum number of base-pair changes required to edit one RNA secondary structure into another. Symbols with over-bars represent average pairwise values, e.g., $\bar{d_{bp}}$ represents an average *d*_*bp*_ (see, e.g., the definition of CAF below).

Extracting windowed Nef subsequences: Two parameters, the window size (Win, an even integer) and the skip size (Skip), were adjustable. Positions of the form *Window*/2 + *k* × *Skip*, (*k* = 0,1,…) within Nef alignment positions 1 to 621 provided coordinates for centering subsequences of length *Window* within the aligned Nef. We extended the Nef subsequences on both sides from their center so that each side included *Window*/2 letters of the original Nef sequence on either side. We optimized *Window* and *Skip* on the Training Dataset, minimizing by eye the overall *p*-values from our statistical tests. To facilitate the interpretation of biological features, the center of each Nef subsequence was mapped back into the reference HXB2 sequence.

Capacity for alternative folds (CAF): For each windowed Nef subsequence *s* described above, according to standard thermodynamic models of RNA secondary structure [10, 57–60], the ViennaRNA (version 2.1.9) [56, 61] command RNAsubopt -s -p 500 sampled *M* = 500 secondary structures *s*_*m*_ from the Boltzmann distribution. The command RNAdistance -DF–Xm calculated the base-pair Hamming distance *d*_*bp*_{*s*_*m*_,*s*_*n*_} between distinct sampled secondary structures *s*_*m*_ and *s*_*n*_, (*m*,*n* = 1,2,…,*M*). The CAF for the subsequence *s* is the average base-pair Hamming distance:

$$CAF\equiv \bar{d_{bp}}=\frac{2}{M(M-1)}\sum_{1\leq m<n\leq M}d_{bp}\{s_{m},s_{n}\}$$

Thus, if a subsequence *s* has a large CAF, then on average, its folds contain very diverse base pairs.

Test of statistical significance: The R function wilcox.test calculates two-sided *p*-values for the Mann-Whitney U-tests [62]. The U-test, which corrects for different sample sizes if necessary, evaluated the statistical significance of CAF changes between Historic and Modern Datasets of Nef subsequences. Adjusted *p*-values [63] based on a variant of the Benjamini Hochberg (BH) procedure [64] corrected for multiple tests by providing an upper bound on False Discovery Rates (FDRs).

Selecting regions (R1, R2, and R3) with significant changes in their CAF: Fifty-three (⌈(*NefLength* − *Window*)/*Skip*⌉ = 53) different locations contributed to the comparison of Historic and Modern Datasets for *Window* = 100 nt and *Skip* = 10 nt, the parameters optimized on the Training Dataset. Of the locations, 6/53 showed a statistically significant CAF change between Historic and Modern Datasets, with *p*-values corresponding to a FDR < 0.001 (shown in red in S1 Fig). The six regions overlapped, so we selected the three regions with the smallest *p*-values for further investigation, naming them R1 (112–211), R2 (362–461), and R3 (492–591). R1, R2, and R3 do not overlap, and all other regions overlap with one of them.

Calculating RNAshapes Entropy: RNAshapes (version 2.1.6) [46], command RNAshapes–p calculated the probabilities for abstract RNA shapes. RNAshapes Entropy is the Shannon entropy of the resulting probability distribution (for details, see Section B.1 in S1 Text). Like CAF, RNAshapes Entropy is a measure of RNA plasticity.

Computing RNA secondary structural stability and the most stable conformation: For each RNA subsequence *s*, the ViennaRNA command RNAfold–T 37C calculated the minimum free energy (MFE), which corresponds to the most stable secondary structure. Similarly, using default parameters, the ViennaRNA command centroid_fold calculated the consensus secondary structure, as follows. The ViennaRNA command centroid_fold randomly sampled *M* = 1000 RNA structures *s*_*m*_ from the Boltzmann distribution, listed all base pairs {*bp*_1_…*bp*_*N*_} occurring in at least half (*M*/2 = 500) of the structures *s*_*m*_, and then combined the base pairs {*bp*_1_…*bp*_*N*_} into a single, consensus structure (for details, see Ding et al. [65]). The free energy of the consensus structure is abbreviated as CFE (consensus free energy). To summarize, therefore, the free energy of the most stable structure is the MFE; of the consensus structure, the CFE. Typically, either a lower MFE or a lower CFE can suggest greater stability of the dominant RNA conformation. In passing, we note that according to the gold standard of RNA structural alignments, the CFE typically predicts RNA structure more accurately than the MFE [65].

Clustering RNA secondary structures for a single sequence *s* or a set *S* of sequences: Consider an arbitrary set of RNA secondary structures. The R function pam implements the Partitioning Around Medoids (PAM) algorithm to partition a set of structures into two clusters.

Calculating dominant and alternative structures for a single R2 subsequence: For each R2 subsequence *s*, the ViennaRNA command RNAsubopt -e 3 –s sampled the Boltzmann distribution of secondary structures. The sampled structures were partitioned into two clusters, as described above. The “dominant structure” of the subsequence *s* was its MFE structure (which was the most stable structure in one of the clusters); and its “alternative structure” was the most stable structure in the other cluster (see Section B.2 in S1 Text for details).

Calculating the Dominant-Alternative Hamming Distance (DAHD) for a single R2 subsequence *s*: The DAHD is the base-pair distance (*d*_*bp*_) between the dominant and alternative structures (see Section B.2 in S1 Text for details).

Calculating dominant and alternative R2 structures from a set *S* of R2 subsequences: Each R2 subsequence *s* has a most stable structure. The PAM algorithm partitioned the R2 subsequences into two clusters according to their most stable structures, as described above. The larger cluster is the “dominant cluster” *C*_1_; the smaller, the “alternative cluster” *C*_2_. Each cluster has a medioid, an R2 subsequence within it whose structure has the minimum average base-pair distance *d*_*bp*_ to the other structures. Each medioid therefore provides a convenient representative of its cluster. The dominant cluster has medioid $\bar{C_{1}}$, “the dominant R2 subsequence” (of the set *S* of R2 subsequences), whose most stable structure is “the dominant R2 structure”. Similarly, the medioid $\bar{C_{2}}$ is “the alternative R2 subsequence”, whose most stable structure is “the alternative R2 structure”. (A warning for all that follows: the adjective “dominant” always refers to the dominant cluster of structures.)

Regressing CAF on MFE: Nested linear regression models of CAF on MFE, with and without a variable indicating whether a sequence was Modern, assessed the statistical significance of CAF changes (see Section B.3 in S1 Text for details).

Mutation test: Among the set of R2 subsequences in the Modern Dataset, the dominant (most stable) structure $\bar{C_{1}(Modern\;R2)}$ corresponds to a particular R2 subsequence, which we call the “wildtype”. The R2 subsequences corresponding to the dominant and alternative structures differed in two consecutive nucleotides M1M2 (398,399) and M5M6 (453,454), and two single nucleotides M3 (405) and M4 (447), corresponding to four hypothetical mutants M1M2, M3, M4, and M5M6 of the wildtype sequence. ViennaRNA compared the MFEs and the most stable structures of the mutants to the wildtype.

Back-Translated (BT) Nef sequences: The EMBOSS (version 6.3.1) [66] command backtranseq generated random sets of hypothetical encoding full-length Nef sequences [11], based on back-translation with the HIV-1 codon-usage file at http://www.kazusa.or.jp/codon/. If a gap caused the amino acid corresponding to a real codon to become ambiguous, the randomization replaced the real codon triplet with three gaps. Thus, BT sequences typically had more gaps than real sequences.

RNA-enforced Back-Translated (REBT) subsequence set: To investigate the significance of Modern R2 plasticity, we generated random RNA sequences such that both their secondary structure (hence, RNA-Enforced) and translated residues (hence, “Back-Translated”) would resemble Modern R2 subsequences as much as possible. The random REBT sequences had the same length (100 nt) as the R2 subsequences, and each of 335 Modern R2 subsequences and its most stable structure served as a template for generating a REBT sequence, as follows:

If a pair of nucleotide positions corresponded to a base pair in the most stable structure of the Modern sequence and both positions corresponded to the third codon, then the corresponding random bases in the REBT sequence were also a potential base pair. The empirical distribution of the base pair was p(A,U) = p(U,A) = 0.169, p(C,G) = p(G,C) = 0.273, and p(G,U) = p(U,G) = 0.058, the probabilities being the base pair frequencies in predictions of most stable structures for all 335 Modern R2 sequences. The frequencies are similar to Rfam base pair frequencies, so our results are likely robust for any reasonable choice of empirical base-pair frequencies. The Infernal command esl-alistat listed base pairs in the most stable structure [67].

If a nucleotide position corresponded to an unpaired nucleotide on the most stable structure of the Modern sequence, then the REBT procedure drew the corresponding base at random from a uniform distribution.

All other nucleotide positions in the REBT sequence remained unchanged. The most stable structure generally paired very few third codon positions. Hence, the REBT procedure prefers to randomize base pairs, even if it changes an amino acid.

Pseudoknot prediction: For a given sequence s, PknotsRG (version 1.3) [68] predicted pseudoknots within the RNA structure. For the R2 region only, the option –m predicted pseudoknots within the MFE structure.

Calculating (Doubly) Differenced Relative Entropy: Relative Entropy calculations followed Peleg et al. [11], which uses MFE predictions from full-length Nef RNA sequence. The EMBOSS (version 6.3.1) [66] command backtranseq generated BT random sequences. Section C in S1 Text contains the details of calculating (Doubly) Differenced Relative Entropy ΔΔ*I* from Historic, Modern, and Back-Translated (BT) random sequences.

Predicting RNA G-quadruplexes (locally and globally stable G4): The ViennaRNA command RNAfold -g predicted thermodynamically stable RNA G4 for both the 100-nt long windowed Nef subsequences (locally stable G4) and the full-length Nef RNA sequence (globally stable G4). See Section D in S1 Text and S3 Table for detailed results.

Visualizing structures: The VARNA software [69] provided RNA secondary structure diagrams.

## Results

### Three regions of Nef show noticeable change in RNA plasticity

CAF quantified the RNA plasticity of windowed Nef subsequences in the Test Dataset. For each Nef subsequence of length 100 nt (the Window parameter) at 10 nt intervals along the Nef RNA sequence (the Skip parameter), the corresponding two-sided Mann-Whitney *p*-value compared the CAFs within the Historic and Modern Datasets (an exploratory optimization on the Training Dataset fixed the Window and Skip parameters used on the Test Dataset). All results presented correspond to the Test Dataset from Cotton et al. [44].

S1 Fig gives the complete set of *p*-values comparing the CAFs of all Historic and Modern Nef subsequences. Our focus here is on the three Nef regions with the most statistically significant changes in CAF, namely, the regions R1, R2, and R3 appearing in Table 2.

**Table 2:** Summary of Nef regions with the most significant change in CAF.

Location on Nef (1–621)	Datasets and *p*-values	*d*_*aa*_	CFE	GC	CAF
R1 (112–211)	Historic	4.69 ± 4.70	-5.08 ± 4.10	0.47 ± 0.02	23.21 ± 7.00
	Modern	5.69 ± 4.48	-6.26 ± 4.66	0.48 ± 0.02	20.77 ± 7.48
	*p*-value		8.69x10^‒4^	5.48x10^-2^	**3.26x10**^**‒5**^
	(*q*-value)		(5.76x10^-3^)	(1.12x10^-1^)	(5.75x10^-4^)
R2 (362–461)	Historic	2.73 ± 1.51	-11.90 ± 4.60	0.52 ± 0.02	13.15 ± 7.25
	Modern	3.46 ± 1.59	-10.88 ± 5.07	0.52 ± 0.02	18.88 ± 8.73
	*p*-value		6.01x10^-3^	4.54x10^-1^	**5.44x10**^**‒19**^
	(*q*-value)		(2.22x10^-2^)	(5.32x10^-1^)	(2.89x10^-17^)
R3 (492–591)	Historic	4.45 ± 2.78	-8.07 ± 4.83	0.54 ± 0.02	16.84 ± 6.35
	Modern	5.23 ± 2.52	-5.85 ± 4.39	0.52 ± 0.03	19.20 ± 6.71
	*p*-value		**5.67x10**^**‒10**^	**5.36x10**^**‒13**^	**1.13x10**^**‒6**^
	(*q*-value)		(3.01x10^-8^)	(9.48x10^-12^)	(3.00x10^-5^)

Values with symbol ± denote mean ± standard deviation. The *p*-values in bold are significant at FDR levels 0.001. FDR calculations were performed separately for each measure of CFE, GC, and CAF.

Table 2 quantifies some important sequence and structure features of the R1, R2, and R3 regions in the Historic and Modern Datasets. The Methods section describes our windowed Nef subsequences. Within each window (i.e., within each set of alignment columns), the average Hamming amino acid distance $\bar{d_{aa}}$ between translated pairs of subsequences quantified the amino acid diversity (the opposite of its conservation). The average of $\bar{d_{aa}}$ over all windows was $\hat{d_{aa}}(Historic)=3.33$ in the Historic Dataset and $\hat{d_{aa}}(Modern)=4.54$ in the Modern Dataset (see Section A in S1 Text). Table 2 shows that in both datasets, the amino acid diversity of R2 was lower than in its average over windowed subsequences (i.e., $2.73=\bar{d_{aa}}(Historic\;R2)<\hat{d_{aa}}(Historic)=3.33$ and $3.46=\bar{d_{aa}}(Modern\;R2)<\hat{d_{aa}}(Modern)=4.54$); the amino acid diversity of R1 and R3, higher. Table 2 also shows that R2 had a lower CFE (i.e., a more stable consensus RNA structure) than R1 and R3. In fact, when we varied the Window parameter (subsequence length) from 100 nt to values between 75 nt to 150 nt, both Historic and Modern Nef sequences in the Training Dataset consistently had the lowest CFE in regions overlapping R2, indicating that R2 contains the most stable local secondary structures within Nef.

In Table 2, the GC compositions of R1 and R2 in particular do not change much from Historic to Modern Datasets, so their GC composition is unlikely to influence directly the other structural features in Table 2.

### RNA plasticity in region R2 increased significantly from Historic to Modern Datasets

#### Modern R2 sequences have gained RNA plasticity as measured by CAF

Overall, the CAF increase from Historic to Modern Datasets was most significant in R2 (*q*-value = 2.89x10^-17^); in contrast to other features such as GC composition and CFE, which changed less in R2 than in R1 or R3. The GC composition of R2 was similar in both Historic and Modern Datasets (0.52 ± 0.02, Table 2). Moreover, at the FDR threshold of 0.01, R2 displayed no significant differences in either GC composition or CFE between the Historic and Modern Datasets. Notably, therefore, the R2 CAF increase from Historic to Modern Datasets lacks a co-association with changes in CFE or GC composition.

#### Other measures confirm increased RNA plasticity in Modern R2

Additional procedures on R2 confirmed its increased RNA plasticity (see the Materials and Methods, and Section B in S1 Text for details). First, the Dominant-Alternative Hamming Distance (DAHD) counted the base-pair differences between structures (cluster medioids) representative of dominant and alternative RNA conformations of each R2 subsequence [53, 54]. Second, the software RNAshapes (version 2.1.6) [46] helped calculate RNAshapes Entropy, yet another, but very different measure of RNA plasticity and alternative folding. Third, an analysis regressing CAF on the MFE quantified the dependency of the increased CAF (as a measure of RNA plasticity) on changes in the MFE (as a measure of RNA stability). The analysis also compared the regression to a similar regression using Simian Immunodeficiency Virus homologs taken from *Pan troglodytes troglodytes* (SIVcpz.ptt), miRNAs, and TPP riboswitches. Genbank [70, 71] provided 5 SIVcpz.ptt sequences [72]. The NCBI/BLAST/TBLASTN tool [73] then extracted regions similar to HIV-1 Nef R2. Rfam [74] provided the miRNA and TPP riboswitch sequences. Like the CAF analysis, the DAHD and RNAshapes Entropy of Modern R2 were higher than those of the Historic R2 (comparing corresponding values in rows “Modern” and “Historic” in Table 3 and also RNAshapes Entropy bar plots in S2 Fig). Regression analyses also supported an increased RNA plasticity in Modern R2 where the F-statistic derived from ANOVA was 82.87 (p = 2.2 x10^‒16^) (see S3 Fig for regression plots). Section B in S1 Text provides extensive detail on the above tests.

**Table 3:** Measures of diversity and plasticity for Modern, REBT (random) Modern, and Historic R2 sets.

R2	$\bar{d_{nt}}$	$\bar{d_{aa}}$	$\bar{d_{bp}}$	MFE	CAF	DAHD	RNAshapes Entropy
Modern	8.30	3.46	38.55	-28.67 ± 4.04	18.88 ± 8.73	30.05 ± 18.11	0.51 ± 0.57
REBT Modern	20.51	4.09	41.43	-32.81 ± 4.07	13.93 ± 6.94	19.55 ± 16.35	0.36 ± 0.51
Historic	4.59	2.73	26.14	-29.49 ± 4.17	13.15 ± 7.25	19.32 ± 16.27	0.20 ± 0.41

#### Modern R2 has more plasticity than random RNA

Column $\bar{d_{aa}}$ of Table 3 shows that although the amino acid diversity ($\bar{d_{aa}}$) of R2 was lower than that of R1 or R3, it was greater in Modern R2 than in Historic R2. We examined possible relationships between amino acid diversity and RNA plasticity with a set of RNA-Enforced-Back-Translated (REBT) random sequences, as follows. For each Modern R2 subsequence, the REBT Modern Dataset contained a random subsequence matched to it by length (100 nt), amino acid sequence, and RNA structural features (see the Materials and Methods). In Table 3, the rows correspond to Modern, REBT Modern, and Historic Datasets of R2 subsequences. The columns correspond to various quantities associated with the sets of R2 subsequences, given as mean ± standard deviation where possible. Although the REBT Modern R2 subsequences had higher nucleotide ($\bar{d_{nt}}$), amino acid ($\bar{d_{aa}}$), and structure ($\bar{d_{bp}}$) diversity than real Modern R2 subsequences, they had lower RNA plasticity (as measured by CAF, DAHD, or RNAshapes Entropy) than real Modern sequences (e.g., 13.93 < 18.88 for CAF).

### Most stable structures of R2 fall naturally into two clusters, the dominant cluster and the alternative cluster

Consider each of the Historic and Modern Datasets in turn. Each R2 subsequence in the Dataset has a most stable structure (its predicted MFE structure). For each Dataset, the structures partitioned naturally into two clusters (i.e., the optimal clustering index under *d*_*bp*_ equaled 2). The larger cluster (which we examine first) we called the dominant cluster; the smaller, the alternative cluster. We chose a “dominant R2 structure” to represent the dominant cluster (the cluster medioid: see the Materials and Methods; also, S1 Table). The dominant R2 structure corresponds to an R2 subsequence, which for brevity and consistency with our other terminology, we call the “dominant R2 subsequence” (but see the warning about terminology in Methods subsection “Calculating dominant and alternative R2 structures from a set *S* of R2 subsequences”).

Substitute “alternative” for “dominant” throughout to obtain analogous definitions of “alternative R2 subsequence” and “alternative R2 structure”.

The dominant R2 structure was the same in both Historic and Modern Datasets, whereas the alternative R2 structure in the Historic Dataset differed by a single base pair from its Modern counterpart. The size of the alternative cluster increased significantly from the Historic to the Modern Dataset, from 40/335 to 101/335 (two-tailed Fisher Exact p = 8.5x10^‒9^).

#### In the Modern Dataset, the RNA structures predicted from the dominant R2 subsequence and from its full-length Nef sequence share features (and similarly, for the alternative R2 subsequence)

Until further notice, consider only the Modern Dataset. If an RNA secondary structure predicted from a full-length sequence shares features with the structure predicted from a subsequence, the agreement increases confidence in the features. With this agreement in mind, define “the dominant Nef sequence” as the full-length Nef sequence (Nef coordinates 1–621) containing the dominant R2 subsequence. The dominant Nef sequence has a most stable RNA structure, called “the dominant Nef structure”. Define similarly “the alternative Nef sequence” and “the alternative Nef structure”. S4 Fig compares the dominant and alternative Nef structures to each other and to the dominant and alternative R2 structures. In S4 Fig, the dominant Nef structure contains the hairpin-like structure called P0 (in blue), as do both the dominant and alternative R2 structures. S4(A) and S4(C) Fig show that the dominant R2 structure shares features with the dominant Nef structure, with both containing both P0 and P1 hairpins. Furthermore, S4(B) and S4(D) Fig show that the alternative R2 structure shared similarities with the alternative Nef structure, with both containing the P2 hairpin. The agreements confirm that the hairpins probably have biological functions.

#### Phylogenetic trees argue against an oversampling bias within our datasets

**Fig 1:**
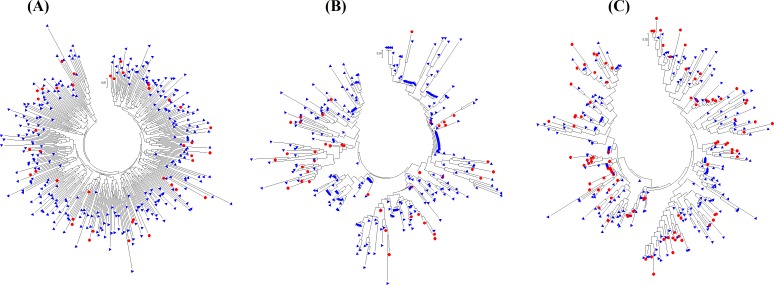
**Phylogenetic trees based on amino acid sequences for (A) Historic full-length Nef, (B) Historic R2, and (C) Modern R2.** Each blue triangle represents a full-length Nef sequence or an R2 subsequence where the most stable R2 structure fell into the dominant cluster; each red circle, into the alternative cluster.

The phylogenetic trees in Fig 1 applied Neighbor-Joining [75] and the *p*-distance method [76] to amino acid distances (*d*_*aa*_) between: (A) the 335 full-length Nef sequences of the Historic Dataset; (B) the 335 Historic R2 subsequences; (C) the 335 Modern R2 subsequences; All analyses were conducted in MEGA6 [77]. Each blue triangle indicates an amino acid sequence whose R2 subsequence had a most stable RNA structure in the dominant cluster; each red circle, in the alternative cluster. In the Historic Dataset, a few R2 subsequences in the dominant cluster generated similar amino acid sequences (see Fig 1(B), occasional concentrations of blue triangles). The similar amino acid sequences dispersed, however, in a phylogenetic tree based on full-length Nef sequences (see Fig 1(A)). The dispersion argues against biases from oversampling any clade in the Historic Dataset. In the Modern Dataset, R2 subsequences displayed no noticeable concentrations of either blue triangles or red circles (see Fig 1(C)). Thus, oversampling biases within our datasets appear unlikely.

#### Five or six nucleotide mutations in R2 can cause a subsequence’s most stable structure to switch between the dominant and alternative structures

The tree-editing distance (*d*_*te*_) between dominant and alternative R2 structures for the Modern Dataset was 82; the base pair distance (*d*_*bp*_), 51 (see S1 Table); the nucleotide distance (*d*_*nt*_) between the dominant and alternative R2 subsequences, 6. (The corresponding figures for the Historical Dataset were *d*_*te*_, 82; *d*_*bp*_, 50; *d*_*nt*_, 5.) For random RNA sequences of length 100 nt, Fig 3 of Schuster et al. [45] shows that the average tree-editing distance (*d*_*te*_) between pairs was about 35, whereas the nucleotide distance (*d*_*nt*_) usually exceeded 20. Thus, the distances between the dominant and alternative R2 structures differ quantitatively from distances between random sequences.

#### The switch between dominant and alternative R2 structures depends mostly on synonymous nucleotide changes

**Fig 2:**
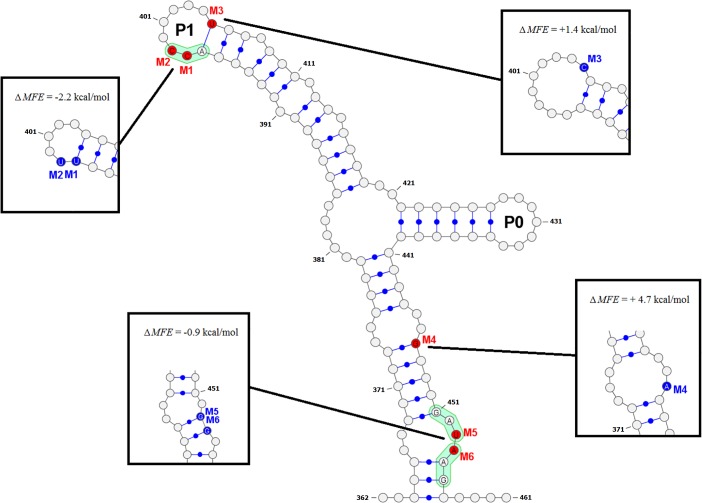
Dominant (wildtype) secondary structure for R2 and structural variations corresponding to hypothetical mutants. The green curves enclose codon triplets. The four rectangle insets show local rearrangements of the RNA secondary structures resulting from four mutations. Mutants are labeled in blue and designated M1M2, M3, M4, and M5M6. Major secondary structural changes caused by mutations are shown inside corresponding squares.

Fig 2 displays the six point mutations converting the dominant R2 subsequence into the alternative R2 subsequence. (See *d*_*nt*_ in the previous paragraph.) As described in the Materials and Methods, the alternative R2 subsequence corresponded to four hypothetical mutants of the dominant “wildtype” sequence: consecutive-base mutants (M1M2, M5M6) and two single nucleotide mutants (M3, M4). Of these, only M3 and M4 are synonymous mutations. The codon containing M1M2 may reflect HLA restrictions [44].

Let ΔMFE denote a mutant MFE minus the wildtype MFE. On one hand, mutations M1M2 in codon (397,398,399) and M5M6 in codons (451,452,453), and (454,455,456) stabilized the dominant structure (ΔMFE = ‒2.2 kcal/mol for M1M2; ΔMFE = ‒0.9 kcal/mol for M5M6). M1M2 also stabilized the important hairpin P1. On the other hand, the synonymous mutations M3 and M4 destabilized it (ΔMFE = +1.4 kcal/mol and + 4.7 kcal/mol, respectively), apparently by unwinding stems.

#### The Alternative conformation for R2 may contain pseudoknots

Pseudoknots are a frequent type of base pairing in RNA structures. Because the secondary structure models in ViennaRNA do not predict pseudoknots, we used PknotsRG (version 1.3) [68] to assess pseudoknot formation in all Historic and Modern R2 subsequences (see S2 Table). PknotsRG predicted no pseudoknots in the dominant R2 structure, but it did predict three pseudoknots in the alternative R2 structure (see S5 Fig). Apart from the pseudoknots, the alternative R2 structure prediction was consistent with that of ViennaRNA. The synonymous change at M3 (mentioned above, at Nef coordinate 405) contributed to pseudoknots (see the black square in S5 Fig).

### The region between R2 and R3 has increased base-pair variability

Following Peleg et al. [11], each full-length Nef RNA sequence yielded a most stable secondary structure in a dot-bracket representation (dots, and left and right parentheses). For four datasets (Historic and Modern, and the corresponding random RNA back-translations preserving protein sequences), background frequencies of dots and brackets were noted (see Section C in S1 Text). Then, we aligned all full-length Nef RNA sequences in the Historic and Modern Datasets together. For each of the four datasets, RNA structural diversity within each alignment column was quantified by the Relative Entropy (***I***) of the frequencies of its dot-bracket characters relative to the background frequencies (with all gap characters ignored). In each column, subtracting the Relative Entropy for the random sequences from that for the real sequences yielded the Differenced Relative Entropy at each aligned position, which accounts for the effect of random sequence variations on structure. Then, subtracting the difference for the Historic Dataset from the difference for the Modern Dataset yielded the (Doubly) Differenced Relative Entropy, denoted by ΔΔ*I*. At each position, ΔΔ*I* represents the change in RNA structural diversity from Historic to Modern Dataset (see Section C in S1 Text for details). The diversity peaked at Positions 476, 482, and 485, i.e., 15 nt downstream of R2 and 15 nt upstream of R3 (see S6 Fig). Although the position of the ΔΔ*I* peaks do not match the previous study exactly, our analyses showed that the known (historic) structural diversity near R2 [11] increased even further from the Historic Dataset to the Modern Dataset.

### Modern R1 contains significantly fewer stable RNA G-quadruplexes than Historic R1

G4s are abundant structural elements in both RNA and DNA, and simple energy models permit RNA secondary structure programs to predict them [78] (see Section D in S1 Text and S3 Table for details). Both local prediction (on 100-nt subsequences) and global prediction (on the full-length Nef RNA sequence) suggested that the positions of both locally and globally stable G4s are highly conserved within and between Historic and Modern sequences, particularly at two locations: 27–39 and 186–200. Locally stable G4s at location 186–200 decreased significantly from the Historic to the Modern Dataset (257/335 to 137/335, p = 3.0x10^‒21^, 76.7% to 40.9%) as did their corresponding globally stable G4 predictions (187/335 to 89/335, p = 1.6x10^‒14^, 55.8% to 26.6%). G4s at location 27–39 displayed a similar (but non-significant) trend (see S3 Table).

## Discussion and Conclusions

**Fig 3:**
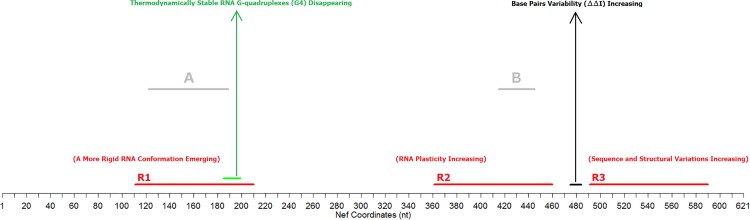
Mapping of this work’s major results regarding Nef RNA. Regions (R1, R2, and R3) in red correspond to locations with significant changes in CAF when comparing Historic and Modern HIV-1 Nef sequences. (See Table 2 for exact coordinates.) The green region (sites 186–200) displays significantly reduced stable G4s. The region in black (sites 476–485) showed high diversity (quantified by the Doubly Differenced Relative Entropy ΔΔ*I*). The grey segment labeled **A** (segment 123–190) refers to the region coinciding with R1 identified by Westerhout et al. [34]; the gray label **B** points to segment 416–446, referred to by Peleg et al. [11]. In Fig 3, the term “Sequence and Structural Variations Increasing” refers to the observed simultaneous changes in the GC composition, CFE, and CAF of Modern R3.

To discover biologically important structural changes in Nef RNA during the evolution of the North American HIV epidemic, we compared two sets of HIV-1 sequences from untreated patients (Historic and Modern) and identified RNA changes in plasticity, quantified here as the capacity for alternative folds (CAF). In addition to plasticity and local RNA structure, our statistical analysis of RNA changes examined pseudoknots and G-quadruplexes, as well as the relative entropies relevant to structural diversity. Fig 3 summarizes relevant results about the Nef gene by others and by the present work.

Many studies focus on the Nef protein, with its subtle and complex activities in viral infectivity. The function of the Nef protein complicates and therefore must temper any interpretation of evolutionary changes in RNA structure. The Nef gene does contain occasional conserved protein regions, however, where protein function is unlikely to drive systematic changes in the Nef RNA. Caveats aside, in moving from the Historic to the Modern Dataset of HIV sequences, significant differences in the 621 nt of Nef RNA included fewer G4s, more pseudoknots, and changes in RNA plasticity depending on the Nef region (less plasticity in R1 (112–211); more plasticity in R2 (362–461) and R3 (492–591). In R3, the GC composition, stability (CFE), and plasticity (CAF) all changed significantly, so correlations complicate any inference about specific selective pressures on R3. Accordingly, our inferences focus on R1 and R2.

### R1 secondary structure became more rigid over time

Modern R1 (region 123–190) displays less nucleotide conservation than most of Nef, and its RNA plasticity and number of thermodynamically stable RNA G4s decrease from the Historic to the Modern Dataset. In fact, RNA base pairs mostly replace the G4s near a conserved region (164–182) targeted by RNAi. An experimental mutation increasing plasticity within R1 permitted an alternative fold that stopped RNAi from binding to the target, enabling Nef RNA to escape RNAi [34]. Here, the emergence of a more stable and rigid RNA secondary structure (decreased CFE and CAF) in R1, where Historic subsequences had an unusually high RNA plasticity (average CAF = 23.21), suggests a recent selective pressure for RNA secondary structural rigidity.

To summarize, although R1 plasticity correlated positively with HIV replication in an experiment, it diminished during the North American HIV epidemic, in seeming contradiction with probable selective pressures on HIV. The biological implications are therefore unclear. Although RNAi-based antiviral therapies might be able to target R1 because of its decreased plasticity, they might also reverse the evolutionary trend to decreased R1plasticity by provoking unwanted escape mutants.

### Unusually strong evidence supports selective pressures on RNA plasticity in R2

RNA in R2 encodes for thirty-two Nef amino acids within the anti-parallel β sheet on β3/β4. In Historic and Modern Datasets, the R2 amino acids are highly conserved (i.e., R2 subsequence pairs have a small *d*_*aa*_). Although the region upstream of R2 sometimes varies in length (see Fig 2 of Reference [72]), an alignment of HIV-1 and SIV_cpz_ Nef proteins displays moderate amino acid conservation in R2. R2 has a low structural free energy (low CFE and MFE), strongly suggesting a locally stable RNA secondary structure, including a highly stable hairpin P0, confirming previous predictions [11].

Despite the amino acid conservation and the RNA structural stability, the most significant change in Nef RNA plasticity occurs in R2. (CAF, DAHD, and RNAshapes Entropy all increased, and regression analysis (Section B.3 in S1 Text) showed that CAF increased, even after accounting for confounding effects from the MFE.) A randomization (REBT) of the R2 subsequence had less plasticity (CAF, DAHD, and RNAshapes Entropy) than real Modern R2 subsequence, suggesting that the high plasticity in R2 may have biological functionality. Moreover, five or six nucleotide mutations can switch the most stable structure for R2 between the dominant and alternative structures, far fewer than the switch requires for random 100 nt sequences. Finally, the Training Dataset from the Los Alamos HIV database showed that the increase in plasticity (CAF) was robust against changes in the Window size (from 50 nt to 150 nt), even for HIV-1 B Nef sequences drawn worldwide.

The relative stability of the dominant and alternative R2 structures does not correlate obviously with Nef protein sequence (see Fig 1). In fact, nonsynonymous nucleotide mutations in R2 (M1M2 and M5M6) usually stabilized one RNA conformation, as opposed to increasing plasticity to accommodate both conformations (see Fig 2). Although sparse, the evidence therefore tends to contradict selection at the amino acid level as a cause of increasing R2 plasticity.

#### Apparent diversity of most stable (MFE) structures in the set of R2 subsequences is likely due to the high RNA plasticity at an individual sequence level

Peleg et al. [11] noted (despite the severe limitations of the computations and data available to them) that the most stable structures for full-length Nef sequences fell into two groups, with the highly conserved hairpin P0 within R2 having length either 5 nt or 7 nt. Although not focused on P0 loop-length, our results showed increased abundance of the alternative most stable structure in Modern R2, in agreement with their conclusions about two different structures near R2. Tests of base-pair variability (ΔΔ*I*, Section C in S1 Text) also supported increased variability of the most stable structure near R2. Finally, tests of plasticity (CAF) show a noticeable increase in thermodynamically stable and RNA conformations available to each individual Modern R2 subsequence.

To summarize, both the RNA plasticity of individual R2 subsequences and the diversity of the most stable structures of R2 subsequences increased over time. In agreement with Peleg et al. [11], our results show that there are two possible most stable structures within R2, a dominant structure and a less common alternative structure, and that the most stable structure of each individual R2 subsequence resembles either the dominant structure or the alternative structure. Our computations show in addition, however, that kinetics take each individual R2 subsequence through equilibrium conformations resembling in turn the dominant and alternative structures. The two findings, about stability and kinetics, relate naturally to each other. On one hand, in a set of R2 subsequences with high RNA plasticity, a few nucleotide mutations in each sequence can toggle its most stable structure between two conformations corresponding to the dominant and alternative structures. On the other hand, R2 is in fact highly conserved, with only minor sequence variability. R2 sequence conservation suggests heavy selective pressure, possibly to preserve RNA plasticity, so every individual Nef sequence has alternative folds available to it.

### R2 plasticity can help evade host responses and might even function in viral RNA switching

The increasing plasticity and potential for alternative folds in and near R2 may therefore be an HIV response to selective pressures from its human host. Such pressures may include cellular and viral RNA molecules in HIV-1-host interactions. As a specific example, Nef-derived miR-N367 targets the 3′-UTR of Nef, which includes R2, to regulate HIV-1 transcription and replication [38], and an alternative fold might evade miR-N367. Alternative folds could also operate like bacterial riboswitches. In some bacterial riboswitches, ligand binding causes allosteric conformational changes propagating along RNA like a domino effect. Among other attractive possibilities, HIV might exploit changes in Nef RNA (either directly or allosterically) to regulate reverse transcription, intra-subtype recombination, or even frameshifting during translation.

### Evidence in support of Nef RNA-protein coevolution

The alternative most stable structure for R2 is more frequent in the Modern than Historic Dataset. The transition from the dominant to alternative most stable structure in R2 requires the P1 hairpin to destabilize, which synonymous changes (M3 and M4) facilitate, and nonsynonymous changes (M1M2 and M5M6) impede. Cotton et al. [44] suggest (see their Fig 6) that mutations at codon (397,398,399) preadapt Nef to its interactions with a restricted Human Leukocyte Antigen (HLA). According to our computations, however, the nonsynonymous mutation M1M2 actually stabilizes P1, favoring the dominant most stable structure and reducing plasticity. Our results suggest that in R2, Nef RNA is under selective pressure to accommodate alternative folds (e.g., the alternative most stable structure). The mutation M3 critical to increasing RNA plasticity is synonymous, ruling out selective pressures on the corresponding amino acid and confining adaptation to RNA (not protein). Possibly, the synonymous changes maintain or increase RNA plasticity and the frequency of alternative folds in R2, while permitting compensatory responses to selection pressures from HLA-polymorphisms at the protein level.

### The hairpin P1 within R2 may be an attractive target for antiviral therapeutics

Targeting stable RNA motifs may be a particularly fruitful therapeutic strategy [79]. R2 is the most stable region of Nef RNA. Within R2, the P1 hairpin appears in predictions of the most stable structure for both R2 and full-length Nef RNA, and its sequence may have to accommodate evolutionary pressures on both protein function and RNA plasticity. Furthermore, experiments suggest that certain HIV-1 viruses can evade RNAi [80, 81]. Hence, if increased R2 plasticity helps HIV evade RNAi, then computational predictions about alternative R2 folds may eventually contribute usefully to clinical decisions about RNAi therapies, by identifying evolutionary bottlenecks for HIV sequences that evade RNAi. Moreover, computations suggest that mutation M3 at position 405 in loop of P1 hairpin forms a pseudoknot only in the alternative R2 structure, which would increase RNA plasticity near P1 by stabilizing the alternative over the dominant R2 structure. Many considerations therefore point to the loop of P1 hairpin in R2 as a particularly attractive target for RNAi-based anti-viral therapeutics.

## Supporting Information

S1 Fig Two-sided Mann–Whitney U-test *p*-values at 10-nt intervals, comparing average Hamming distance between 335 Historic and 335 Modern Nef subsequences of length 100 nt. The blue points correspond to FDR not exceeding 0.01; the red, to an FDR not exceeding 0.001. The three bold red line segments correspond to the regions designated R1, R2, and R3.
(PNG)

S2 Fig RNAshapes Entropy histogram of Historic and Modern Nef R2 subsequences. Colors blue, red, green, and black roughly correspond to number of structures 1, 2, 3, and 4, respectively (see Materials and Methods).
(PNG)

S3 Fig MFE vs. CAF linear regression of R2. (A) CAF values of Historic R2 subsequences (335 red data points), Modern R2 subsequences (335 green data points), and SIVcpz.ptt (5 blue data points). Colored lines represent the corresponding linear regression models. Adjusted R-squared value of the linear model CAF~MFE by combining Historic and Modern Datasets (670 data points) was 0.2455. Adjusted R-squared value of the model CAF~MFE+R2 distinguishing Historic and Modern Datasets in the model, was 0.3279. ANOVA test between the two models including and excluding variable R2 gives F-value of 82.87 (p = 2.2 x10^‒16^). (B) CAF values from sequence datasets corresponding to miRNA (19 red data points) and TPP-riboswitch (42 blue data points). Colored lines represent the corresponding linear regression models. Adjusted R-squared value of the model CAF~MFE by combining miRNA and TPP-riboswitch Sets (61 data points) was 0.3112. Adjusted R-squared value of the linear model CAF~MFE+ncRNA distinguishing miRNA and TPP-riboswitch sets, was 0.3199. ANOVA test between the models including and excluding variable ncRNA gives F-value of 1.7588 (p = 0.19). The gray area in each plot shows a 95% confidence band for the linear regression model.
(PNG)

S4 Fig Most stable RNA secondary structures of two representative Modern R2 structures. (A) The dominant R2 structure. (B) The alternative R2 structure. (C) The dominant Nef structure (i.e., the most stable structure of the full-length Nef sequence containing the R2 subsequence whose most stable structure is the dominant R2 structure). (D) The alternative Nef structure (i.e., the most stable structure of the full-length Nef sequence containing the R2 subsequence whose most stable structure is the alternative R2 structure). In A and B, red nucleotides mark the six mutations that switch the most stable structure between the dominant and alternative R2 structures. The conserved hairpin P0 is shown in blue. In C and D, R2 is marked in red, to contrast the structural predictions for the R2 subsequence and the full-length Nef sequence. In C and D, the green circles indicate notable ΔΔ*I* peaks.
(PNG)

S5 Fig Pseudoknot prediction in the alternative R2 structure. The triple pseudoknot is shown with blue arrows. Hairpins P0 (shown in red stripe) and P2 are identical to prediction of the alternative structure. Nucleotide differences from those of the dominant structure are shown in red. Black square shows location of the synonymous nucleotide difference that corresponds to position of mutation M3. PKnotsRG [68] program was used for prediction. Visualization was done using VARNA [69].
(PNG)

S6 Fig (Doubly) Differenced Relative Entropy ΔΔ*I*. Top five values are shown in black dots. Positions 145, 476, 482, 485, and 490. Standard deviation resulting from sampling bias shown in red. Standard deviation value of each position was equal to the square root of sum of the individual variance measures corresponding to the four datasets. See Materials and Methods for more details.
(PNG)

S1 TableClustering statistics of region R2 structures.MFE predictions of R2 sequences were used for clustering (See Materials and Methods). Base-pair Hamming distances between the two medoids of clusters are shown in column $d_{bp}\{\bar{C_{1}(R2)},\bar{C_{2}(R2)}\}$.(DOCX)

S2 Table Prediction of locally stable pseudoknots in R2. Total No. of predicted pseudoknots shows the number of pseudoknot pairs observed in a total of 335 sequences in each set. Locations of pseudoknots varied. The location of predicted pseudoknots in the alternative structure was conserved between the Historic and Modern counterparts. PKnotsRG [68] program was used for prediction.
(DOCX)

S3 Table Location and frequency of locally and globally stable RNA G-quadruplexes (G4) in the Historic and Modern Nef RNA datasets. Predictions made using RNAfold–g command from ViennaRNA Software (version 2.1.9) [56]. Rows “Global Historic” and “Global Modern” represent results corresponding to the full-length Nef sequence (globally stable RNA G4s). Rows “Local Historic” and “Local Modern” represent results corresponding to 100 nt windowed Nef subsequences (locally stable RNA G4s) that best surround locations derived from the global predictions. The blue column shows the location of the RNA G4 that had a noticeable decrease in frequency in Modern sequences.
(DOCX)

S1 Text Supplementary Materials. Section A: The average of Hamming amino acid distance over all 100-nt windows of Nef sequences. Section B: Additional Tests of RNA plasticity in Historic and Modern R2. Section C: Base-pair variability using full-length Nef RNA secondary structures. Section D: Globally and locally thermodynamically stable RNA G-quadruplexes within region R1.
(DOCX)
