# Supplementary figures and images for: Changes in the Plasticity of HIV-1 Nef RNA during the Evolution of the North American Epidemic
Source: PLoS One. 2016 Sep 29;11(9):e0163688. doi: 10.1371/journal.pone.0163688 (PMC5042412; doi:10.1371/journal.pone.0163688)

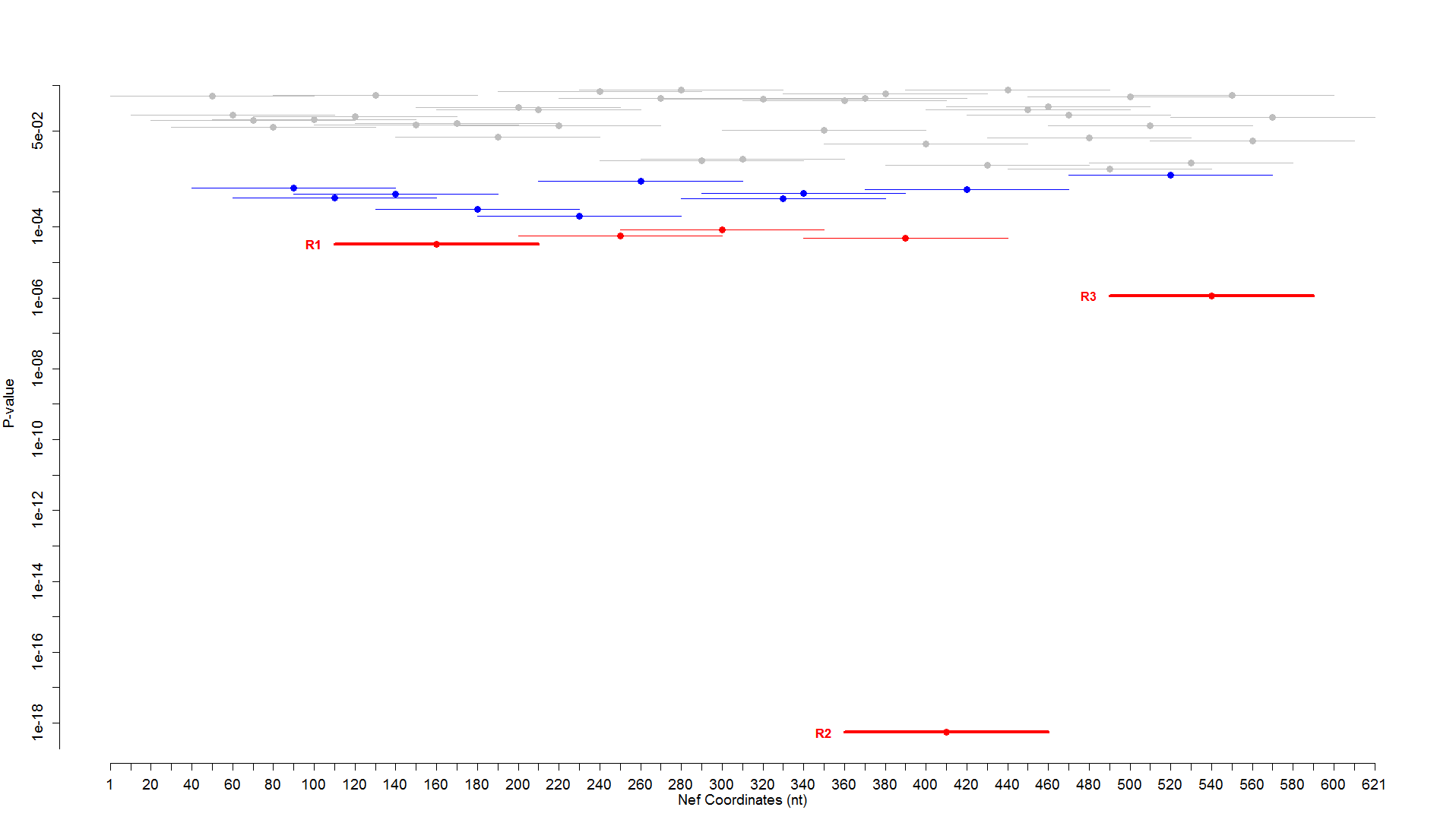

Supplement: S1 Fig — The blue points correspond to FDR not exceeding 0.01; the red, to an FDR not exceeding 0.001. The three bold red line segments correspond to the regions designated R1, R2, and R3. (PNG) [file pone.0163688.s001.png]

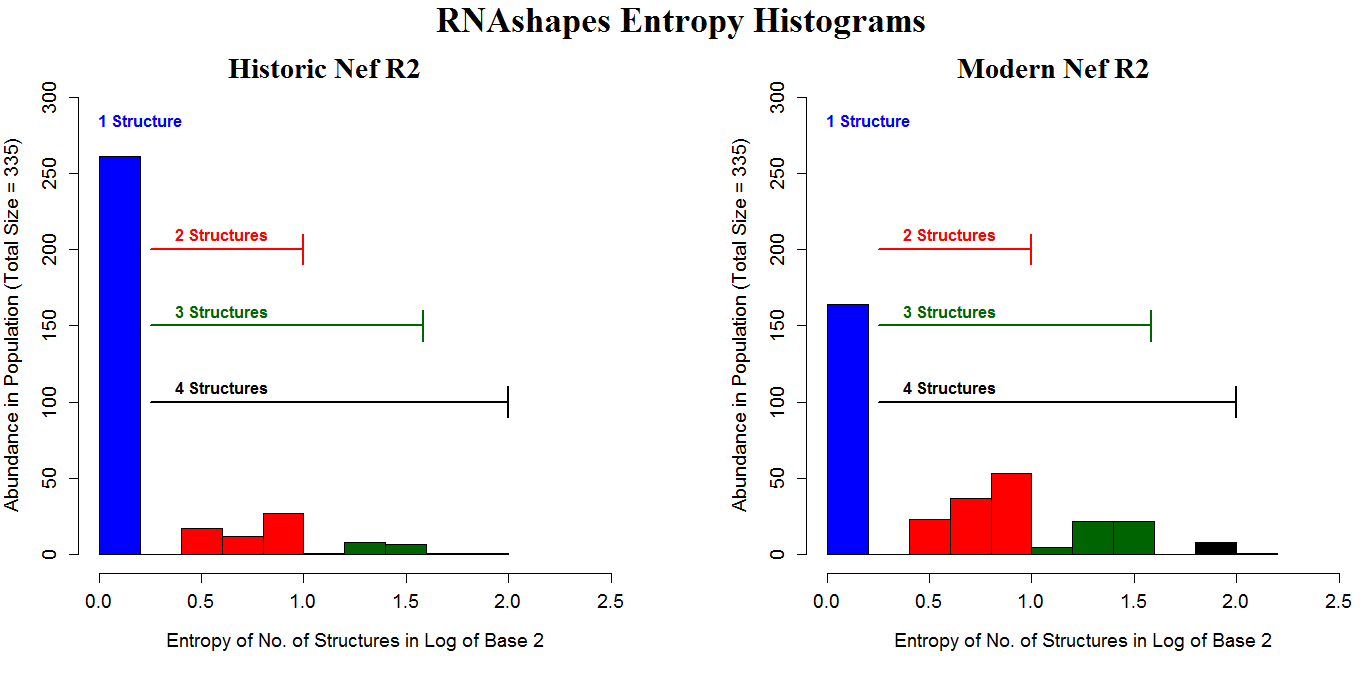

Supplement: S2 Fig — Colors blue, red, green, and black roughly correspond to number of structures 1, 2, 3, and 4, respectively (see Materials and Methods). (PNG) [file pone.0163688.s002.png]

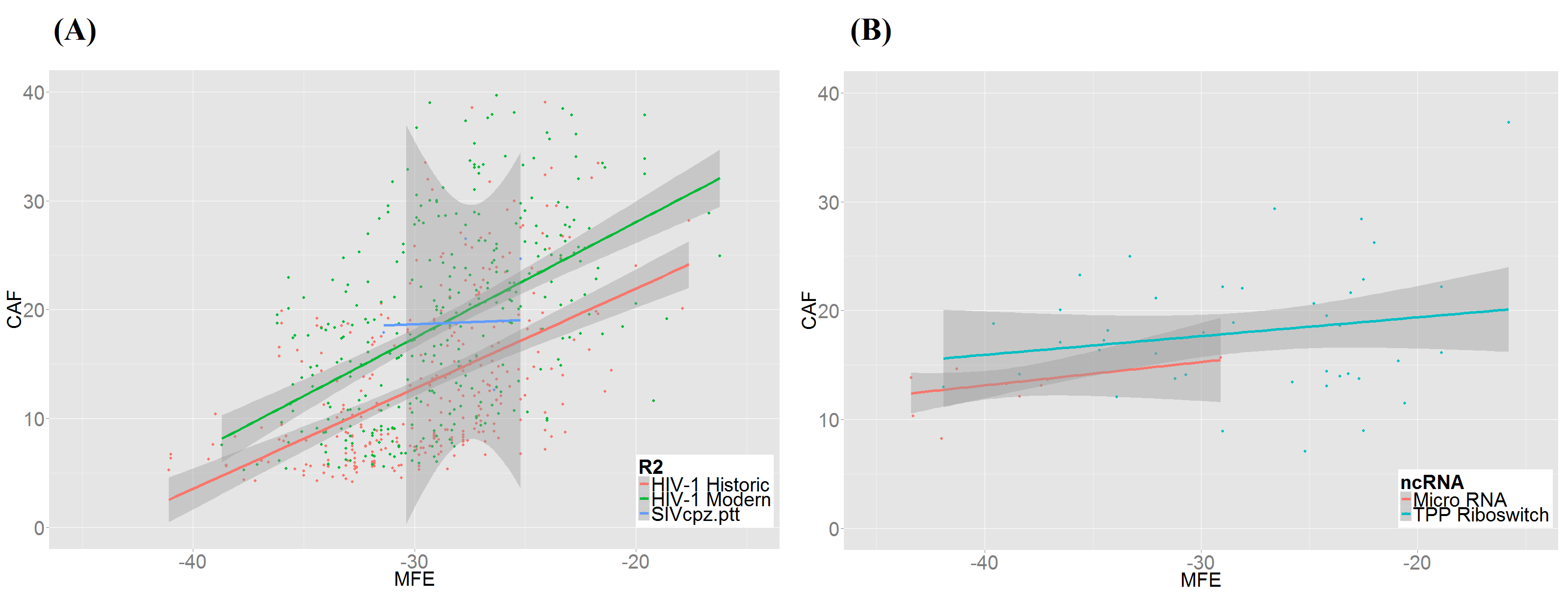

Supplement: S3 Fig — (A) CAF values of Historic R2 subsequences (335 red data points), Modern R2 subsequences (335 green data points), and SIVcpz.ptt (5 blue data points). Colored lines represent the corresponding linear regression models. Adjusted R-squared value of the linear model CAF~MFE by combining Historic and Modern Datasets (670 data points) was 0.2455. Adjusted R-squared value of the model CAF~MFE+R2 distinguishing Historic and Modern Datasets in the model, was 0.3279. ANOVA test between the two models including and excluding variable R2 gives F-value of 82.87 (p = 2.2 x10‒16). (B) CAF values from sequence datasets corresponding to miRNA (19 red data points) and TPP-riboswitch (42 blue data points). Colored lines represent the corresponding linear regression models. Adjusted R-squared value of the model CAF~MFE by combining miRNA and TPP-riboswitch Sets (61 data points) was 0.3112. Adjusted R-squared value of the linear model CAF~MFE+ncRNA distinguishing miRNA and TPP-riboswitch sets, was 0.3199. ANOVA test between the models including and excluding variable ncRNA gives F-value of 1.7588 (p = 0.19). The gray area in each plot shows a 95% confidence band for the linear regression model. (PNG) [file pone.0163688.s003.png]

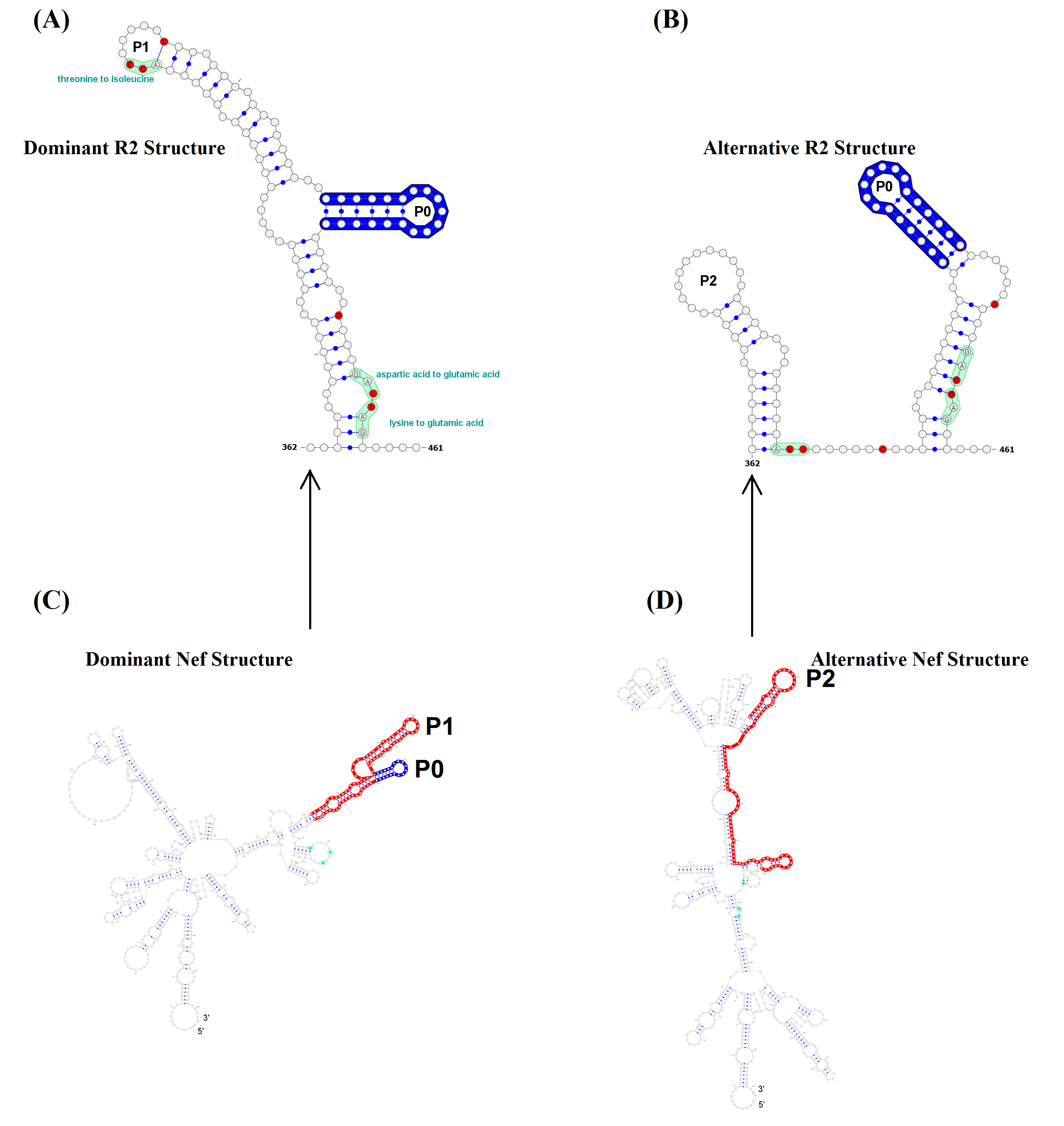

Supplement: S4 Fig — (A) The dominant R2 structure. (B) The alternative R2 structure. (C) The dominant Nef structure (i.e., the most stable structure of the full-length Nef sequence containing the R2 subsequence whose most stable structure is the dominant R2 structure). (D) The alternative Nef structure (i.e., the most stable structure of the full-length Nef sequence containing the R2 subsequence whose most stable structure is the alternative R2 structure). In A and B, red nucleotides mark the six mutations that switch the most stable structure between the dominant and alternative R2 structures. The conserved hairpin P0 is shown in blue. In C and D, R2 is marked in red, to contrast the structural predictions for the R2 subsequence and the full-length Nef sequence. In C and D, the green circles indicate notable ΔΔI peaks. (PNG) [file pone.0163688.s004.png]

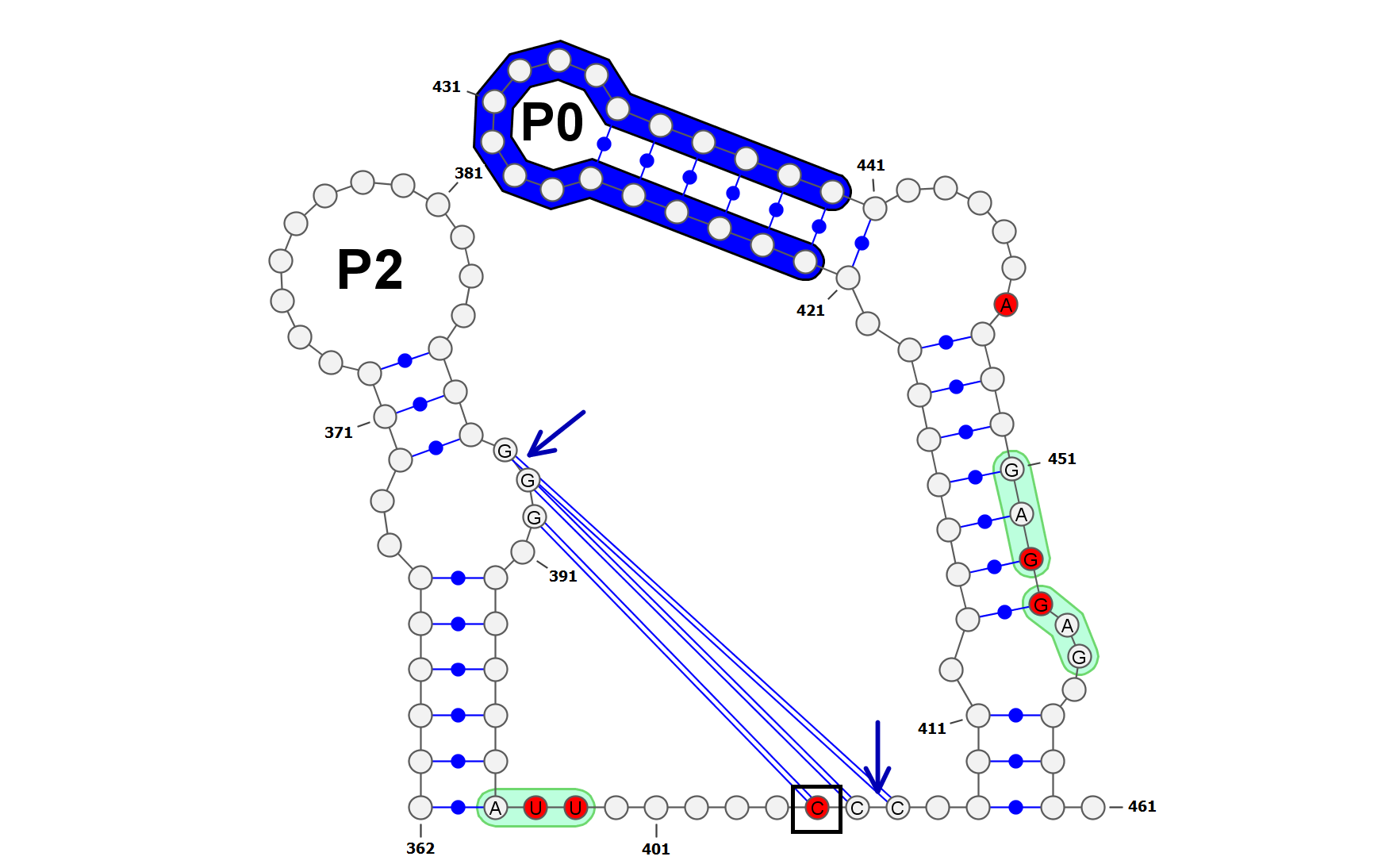

Supplement: S5 Fig — The triple pseudoknot is shown with blue arrows. Hairpins P0 (shown in red stripe) and P2 are identical to prediction of the alternative structure. Nucleotide differences from those of the dominant structure are shown in red. Black square shows location of the synonymous nucleotide difference that corresponds to position of mutation M3. PKnotsRG [68] program was used for prediction. Visualization was done using VARNA [69]. (PNG) [file pone.0163688.s005.png]

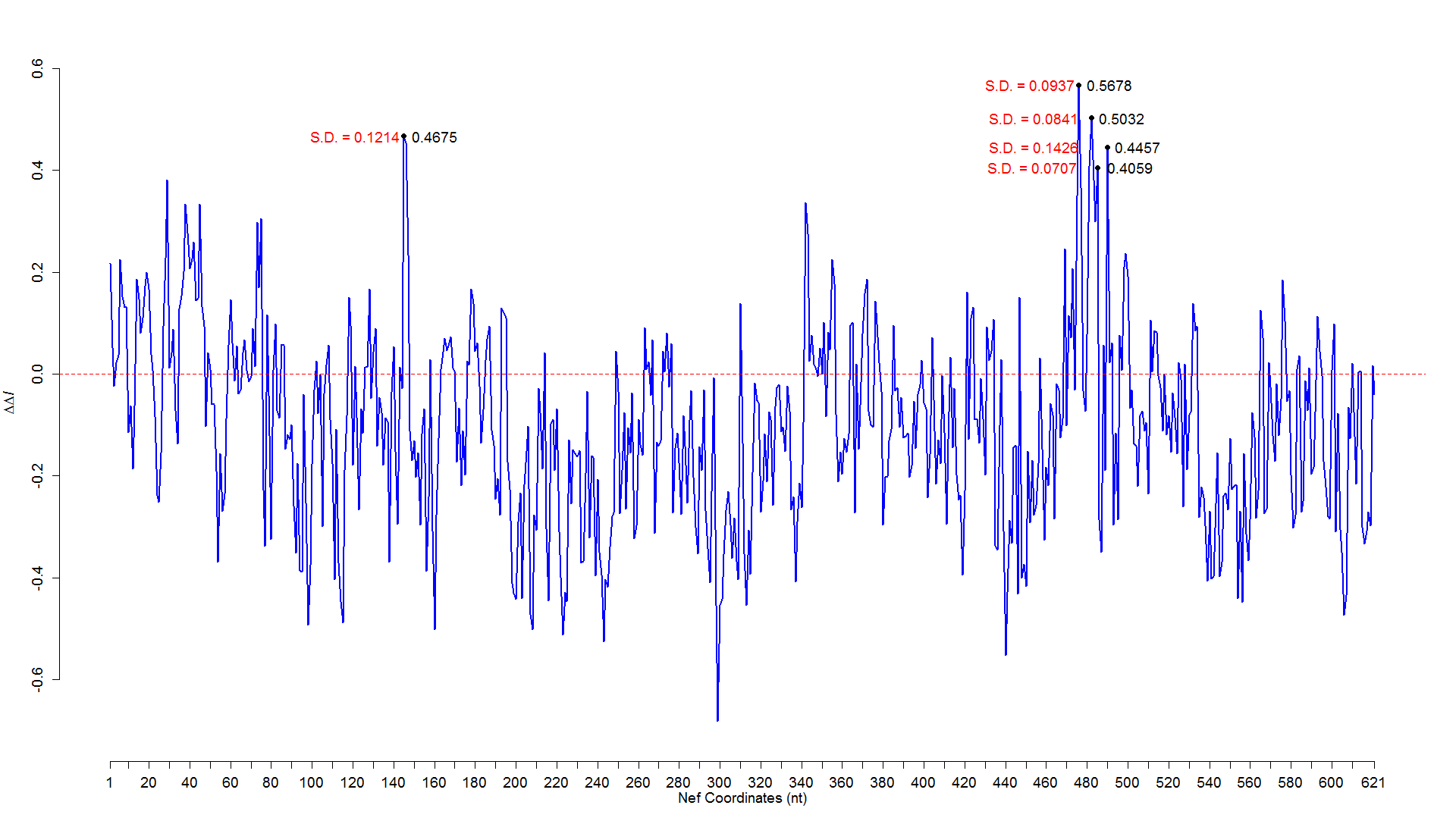

Supplement: S6 Fig — Top five values are shown in black dots. Positions 145, 476, 482, 485, and 490. Standard deviation resulting from sampling bias shown in red. Standard deviation value of each position was equal to the square root of sum of the individual variance measures corresponding to the four datasets. See Materials and Methods for more details. (PNG) [file pone.0163688.s006.png]
