# Supplementary material for: Changes in the Plasticity of HIV-1 Nef RNA during the Evolution of the North American Epidemic
Source: PLoS One. 2016 Sep 29;11(9):e0163688. doi: 10.1371/journal.pone.0163688 (PMC5042412; doi:10.1371/journal.pone.0163688)
Supplement: S1 Table — MFE predictions of R2 sequences were used for clustering (See Materials and Methods). Base-pair Hamming distances between the two medoids of clusters are shown in column dbp{C1(R2)¯,C2(R2)¯}. (DOCX) [file pone.0163688.s007.docx]

| $\boldsymbol{R}\boldsymbol{2}$ | $\boldsymbol{Size}\left[ \begin{matrix} \boldsymbol{C}_{\boldsymbol{1}}\boldsymbol{(R}\boldsymbol{2)} & \boldsymbol{C}_{\boldsymbol{2}}\boldsymbol{(R}\boldsymbol{2)} \end{matrix} \right]$ | $\boldsymbol{AveDist}\left[ \begin{matrix} \boldsymbol{C}_{\boldsymbol{1}}\boldsymbol{(R}\boldsymbol{2)} & \boldsymbol{C}_{\boldsymbol{2}}\boldsymbol{(R}\boldsymbol{2)} \end{matrix} \right]$ | $\boldsymbol{d}_{\boldsymbol{bp}}\left\{ \bar{\boldsymbol{C}_{\boldsymbol{1}}\left( \boldsymbol{R}\boldsymbol{2} \right)}\boldsymbol{,}\bar{\boldsymbol{C}_{\boldsymbol{2}}\left( \boldsymbol{R}\boldsymbol{2} \right)} \right\}$ |
| --- | --- | --- | --- |
| Modern | $\left[ \begin{matrix} 234 & 101 \end{matrix} \right]$ | $\left[ \begin{matrix} 22.68 & 20.49 \end{matrix} \right]$ | 51 |
| REBT Modern | $\left[ \begin{matrix} 228 & 107 \end{matrix} \right]$ | $\left[ \begin{matrix} 25.93 & 22.55 \end{matrix} \right]$ | 54 |
| Historic | $\left[ \begin{matrix} 295 & 40 \end{matrix} \right]$ | $\left[ \begin{matrix} 12.73 & 18.28 \end{matrix} \right]$ | 50 |
