# Supplementary material for: Changes in the Plasticity of HIV-1 Nef RNA during the Evolution of the North American Epidemic
Source: PLoS One. 2016 Sep 29;11(9):e0163688. doi: 10.1371/journal.pone.0163688 (PMC5042412; doi:10.1371/journal.pone.0163688)
Supplement: S2 Table — Total No. of predicted pseudoknots shows the number of pseudoknot pairs observed in a total of 335 sequences in each set. Locations of pseudoknots varied. The location of predicted pseudoknots in the alternative structure was conserved between the Historic and Modern counterparts. PKnotsRG [68] program was used for prediction. (DOCX) [file pone.0163688.s008.docx]

| $\boldsymbol{R}\boldsymbol{2}$ | **No. of sequences with**  **Pseudoknot predictions** | **Total No. of predicted**  **pseudoknots in Data** | **No. of Predicted**  **pseudoknots in Dominant Conformation** | **No. of Predicted**  **pseudoknots in Alternative Conformation** |
| --- | --- | --- | --- | --- |
| Modern | 131/335 | 1192 | 0 | 3 |
| REBT Modern | 108/335 | 1030 | 0 | 0 |
| Historic | 77/335 | 742 | 0 | 3 |
