# Supplementary material for: Changes in the Plasticity of HIV-1 Nef RNA during the Evolution of the North American Epidemic
Source: PLoS One. 2016 Sep 29;11(9):e0163688. doi: 10.1371/journal.pone.0163688 (PMC5042412; doi:10.1371/journal.pone.0163688)
Supplement: S3 Table — Predictions made using RNAfold–g command from ViennaRNA Software (version 2.1.9) [56]. Rows “Global Historic” and “Global Modern” represent results corresponding to the full-length Nef sequence (globally stable RNA G4s). Rows “Local Historic” and “Local Modern” represent results corresponding to 100 nt windowed Nef subsequences (locally stable RNA G4s) that best surround locations derived from the global predictions. The blue column shows the location of the RNA G4 that had a noticeable decrease in frequency in Modern sequences. (DOCX) [file pone.0163688.s009.docx]

| **Location (nt)** | **4-15** | **27-39** | **81-91** | **186-200** | **283-295** | **456-466** |
| --- | --- | --- | --- | --- | --- | --- |
| Global Historic | 0/335 | 21/335 | 1/335 | 187/335 | 1/335 | 1/335 |
| Global Modern | 1/335 | 18/335 | 1/335 | 89/335 | 0/335 | 0/335 |
| Local Historic | 0/335 | 25/335 | 1/335 | 257/335 | 4/335 | 3/335 |
| Local Modern | 0/335 | 21/335 | 2/335 | 137/335 | 2/335 | 1/335 |
