# Supplementary material for: Changes in the Plasticity of HIV-1 Nef RNA during the Evolution of the North American Epidemic
Source: PLoS One. 2016 Sep 29;11(9):e0163688. doi: 10.1371/journal.pone.0163688 (PMC5042412; doi:10.1371/journal.pone.0163688)
Supplement: S1 Text — Section A: The average of Hamming amino acid distance over all 100-nt windows of Nef sequences. Section B: Additional Tests of RNA plasticity in Historic and Modern R2. Section C: Base-pair variability using full-length Nef RNA secondary structures. Section D: Globally and locally thermodynamically stable RNA G-quadruplexes within region R1. (DOCX) [file pone.0163688.s010.docx]

Supplementary Materials

# Section A: The average of Hamming amino acid distance over all 100-nt windows of Nef sequences

## Average Hamming amino-acid distances for the full-length Nef sequence $\bar{\boldsymbol{d}_{\boldsymbol{aa}}}\boldsymbol{(1-621)}$ were 20.7 (Historic) and 28.2 (Modern), so calibrating with a null hypothesis that all amino acids in Nef are equally conserved, the average of amino-acid distance between pairs of 100-nt-windowed Nef subsequences are $\hat{\boldsymbol{d}_{\boldsymbol{aa}}}\boldsymbol{=}\boldsymbol{20.7 \times100/621 = 3.33}$ (Historic) and $\boldsymbol{28.2 \times100/621 = 4.54}$ (Modern).

# Section B: Additional tests of RNA plasticity in Historic and Modern R2

## Section B.1: RNAshapes Entropy as measure of RNA plasticity

RNAshapes (version 2.1.6) [[1](#_ENREF_1)] quantified the dissimilarity of the conformations available to a given RNA sequence, as follows. Given an RNA sequence, the RNAshapes –p command essentially lists the dissimilar abstraction of shapes and their probabilities. RNAshapes Entropy is the Shannon Entropy in bits, calculated from the probabilities of the abstract shapes. Before calculating the Shannon Entropy, we used the cut-off value of 0.1 to filter out structures with low probability. The number of structures above the cut-off realistically quantified the structural alternatives for a given RNA subsequence, but in fact, the cut-off did not change the RNAshapes Entropy much. The tool examined the abstract shapes associated with the subsequence. S2 Fig shows the distribution of RNAshapes Entropy values for Historic and Modern R2 (See Materials and Methods Section). Various colors indicate roughly the likely range for a specific number of structures for the R2 sequence. Much fewer Modern (164) than Historic (261) sequences have only one stable RNA secondary structure (comparing blue bars). The number of subsequences with one fold significantly decreases over time, (from 261/335 to 164/335, two-tailed Fisher Exact p = 7.4x10^‒15^, 78% to 49%). Conversely, the Modern R2 Set contains more subsequences with two, three, or more structures than the Historic R2 Set (See red, green, and black bars, respectively).

## Section B.2: DAHD as a measure of RNA plasticity

MFE Structurecalculations for dominant and alternative structures: A standard procedure [[2](#_ENREF_2), [3](#_ENREF_3)] yielded the alternative structure for the subsequence R2. First, repetitions of the ViennaRNA command RNAsubopt -e 3 –s sampled independent suboptimal secondary structures within 3 kcal/mol (an arbitrary but inclusive cut-off) of the MFE for R2. If the samples exceeded 500, we used only the first 500. After partitioning the structures into two clusters, the minimum free energy within the alternative cluster determined the “alternative structure”$MFE\_Alt(s)$ of the subsequence $s$.

Dominant-Alternative Hamming Distance (DAHD) calculations: The DAHD is Hamming (base pair) difference $d_{bp}\left\{ MFE\left( s \right),MFE\_Alt\left( s \right) \right\}$ between RNA secondary structures corresponding to the overall MFE (which corresponds to the MFE within the dominant cluster) and the MFE of the alternative cluster of the subsequence $s$.

To investigate RNA secondary structural dynamics of Modern R2, we measured the dissimilarity between the RNA secondary structures corresponding to the overall MFE (which is the MFE of the dominant cluster) to the MFE of the alternative cluster. For a given subsequence $s$, call the base-pairing distance $d\left\{ MFE\left( s \right),MFE\_Alt(s) \right\}$ between the MFE and the alternative structure, the “Dominant-Alternative Hamming Distance” (DAHD). (See the Materials and Methods section.) The DAHD value estimates the conformational distance between the dominant and alternative MFE structures, making it a measure of the structural diversity of a given RNA sequence. Table 3 shows that similar to the CAF test, the DAHD values were higher for the Modern R2 compared to both REBT Modern R2 and Historic R2 subsequences (Historic R2). Both CAF and DAHD support the conclusion that Modern R2, on average, had a higher folding diversity than both Historic counterparts and randomly generated sequences, indicating that the conclusion is robust against technical changes in the sampling procedures or derived statistics.

## Section B.3: MFE-CAF regression analyses

Two nested linear regression models evaluated CAF changes within R2, while controlling for stability using the RNA minimum free energy (rather than CFE). The first model was CAF ~ MFE; the second, CAF ~ MFE + X, where X is a scalar variable identifying the set to which the sequence belongs. Because of nesting, the ANOVA F-statistic and its p-value can assess whether CAF differs significantly between two populations. Variants of the regression analyses (e.g., substituting CFE for MFE or using exponential rather than linear regression) yielded similar results.

Although the CFE difference for R2 (two-sided Mann-Whitney p = 6.01x10^-3^, q-value 2.22x10^-2^) is uninteresting at a threshold FDR = 0.01, we sought to assess its possible impact on the CAF by investigating the relationship between secondary structural diversity and stability for individual R2 subsequences. Diversity-stability regression analyses also suggested that Modern R2 subsequences (Modern R2) had significantly higher CAF values than their Historic counterparts. The F-statistic and corresponding p-value derived from ANOVA were 82.87 and Pr(>F) < 2.2 x10^‒16^, respectively (See Materials and Methods Section).

Collection of Simian Immunodeficiency Virus (SIV) Nef sequences corresponding to region R2 from chimpanzees: Our results highlighted the possible importance of the Nef region R2 (the Results Section has the sequence coordinates for R2). To understand our results in a broader context, we drew on a study aligning Nef sequences from HIV-1 in humans and from SIV in chimpanzees [[4](#_ENREF_4)]. The study incidentally noted R2 sequence conservation across species. We retained for our analysis only the five sequences corresponding to the SIV Nef R2 in the chimpanzee species *Pan troglodytes troglodytes* (*P.t.t.*), because they displayed more similarity than other chimpanzee sequences to the HIV-1 group M sequences of interest here. First, we extracted corresponding Nef sequences from Genbank [[5](#_ENREF_5), [6](#_ENREF_6)]. Then, we discarded all but the 100-nt long sequences most closely corresponding to the HIV-1 Nef R2 (according to the NCBI/BLAST/TBLASTN tool [[7](#_ENREF_7)]). To calibrate for the reader the divergence of the resulting 100-nt long sequences denoted here as SIVcpz.ptt, they all had at least 28 out of 32 amino acids in common with an arbitrary Historic Nef sequence. S3 Fig (A) illustrates linear regression prediction curve of CAF values with respect to MFE for R2 of Historic, Modern, and SIVcpz.ptt sets, separately. Although the regression corresponding to SIVcpz.ptt counterparts was closer to that of the HIV-1 Nef Modern sequences, the 0.95 confidence interval shown in gray did not suggest an immediate distinction between SIV and HIV-1 sets. The regressions in S3 Fig (A) indicate that CAFs in Modern R2 are larger than CAFs in Historic R2, regardless of the MFE.

Collection of microRNA and TPP-Riboswitch sequences: We extracted seed sequences for the miRNA families with accession numbers RF00455, RF00981, RF01895, RF01896, RF01897, RF01900, RF01911, RF01916, RF01925, RF01927, RF01938, RF01941, RF01996, RF02000, RF02020 in Rfam [[8](#_ENREF_8)]. Accession numbers were chosen randomly from amongst all other miRNAs. We discarded sequences with lengths (*L*) outside the range 95 ≤ *L* ≤ 105, leaving 19 miRNA sequences. Similarly, the thiamine pyrophosphate (TPP) riboswitches (accession number RF00059 in Rfam) yielded 42 sequences. We chose to examine the TPP riboswitch, because the literature considers it a “typical” riboswitch, and because various computational tools have successfully captured traces of the two alternative structures RNA in its secondary structure space. Neither the miRNAs nor the TPP riboswitches were filtered further (e.g., for similar GC-compositions), since the resulting sets would be too small for our testing purposes. S3 Fig (B) shows regression curves corresponding to miRNAs and TPP riboswitches, separately. The F-statistic and its corresponding p-value were 1.7588 and Pr(>F) = 0.19. S3 Fig (B) shows that TPP riboswitches having two biologically functional alternative secondary structures (two structures) had higher CAF values compared to miRNAs that only have one stable secondary structural conformation (one structure). Therefore, S3 Fig hints at the possibility that R2 might be evolving a functionality involving alternative conformations.

# Section C: Base-pair variability using full-length Nef RNA secondary structures

(Doubly) Differenced Relative Entropy calculations: MFE predictions of the full-length Nef RNA sequence (1-621) helped elucidate the distribution of information along the Nef sequence alignment. Eq. 1 of [[9](#_ENREF_9)] yielded the relative entropy for a given set $S$ of sequences (In contrast to Peleg et al. [[9](#_ENREF_9)], we did not use gap characters in information calculations). Each set of sequences yielded a predicted RNA structure. The empirical base-pair distributions in the Historic and Modern MFE predictions were very similar to those previously found [[9](#_ENREF_9)]. The dot-bracket notation (Stockholm format) for RNA structure uses three characters: ‘.’ denotes an unpaired base, while ‘(‘ and ‘)’ denote a base pair. Given a set $S$ of Nef sequences, let $\hat{I}(S)=\left\{ \begin{matrix} \hat{I}_{1} & \ldots& \hat{I}_{621} \end{matrix} \right\}$, where the relative entropy $\hat{I}_{i}$ (also known as “information content” elsewhere [[9](#_ENREF_9)]) quantifies the diversity of structural features at position $i$ of the Nef region relative to the distribution of structural features within the population of MFE predictions.

Back-Translated (BT) Nef sequences: The EMBOSS (version 6.3.1) [[10](#_ENREF_10)] command backtranseq generated random sets of hypothetical encoding Nef sequences [[9](#_ENREF_9)], based on back-translation with the HIV-1 codon-usage file at <http://www.kazusa.or.jp/codon/>. If a gap caused the amino acid corresponding to a real codon to become ambiguous, the randomization replaced the codon triplet with three gaps. Thus, BT sequences typically had more gaps than real sequences. We defined differenced relative entropy as

$$\Delta\Delta I=\left[ I\left( Modern \right)-I\left( BT Modern \right) \right]-\left[ I\left( Historic \right)-I(BT Historic) \right]$$

Sampling bias was determined evaluating the expectation and variance of the random variable $I_{i}\left( S \right)=\sum_{k} q_{ki}\lg q_{ki}/p_{k}$, where vector $Q_{i}$ is the probability vector of structural features corresponding to position $i$ of the alignment and $P$ is the probability vector of structural features of the complete data. Differences between the actual and the expected values were negligible. Since $\Delta\Delta I$ was the linear combination of different random variables, it was essential that we calculate the overall variance of the estimator for $\Delta\Delta I$. As an approximation, the standard deviation of $\Delta\Delta I$ estimates at every position was taken as the square root of the sum of individual variance values corresponding to the four datasets. Where relevant, all results are represented in bits, i.e., logarithms are base 2.

The $I(S)$ Signals were highly correlated ($\rho=0.89$), suggesting that MFE predictions in the Historic and Modern had generally similar relative entropies. However, Nef sequences were generally more diverse in the Modern Dataset than in the Historic Dataset. Back-Translated (BT) sequences provide some context for the next analysis, because although BT sequences have no sequence or structural constraints other than amino acid sequence, the Differenced Relative Entropy $\Delta I(S)=I\left( S \right)-I(BT S)$ can be informative of position-specific structural behavior within any set $S$ of Historic, Modern, or random sequences (see, e.g., [[9](#_ENREF_9)]). To investigate rates of change in RNA secondary structural features over time, we calculated the (Doubly) Differenced Relative Entropy $\Delta\Delta I$ between Historic and Modern sequences as detailed in the Materials and Methods section and shown for the full-length of Nef RNA S6 Fig

S6 Fig shows the top five $\Delta\Delta I$ values (in black) along with the corresponding positions (145, 476, 482, 485, and 490) and the sampling bias (in red). The third and fourth highest peaks (Positions 145 and 490) had higher sampling bias. Hence, the estimated peaks for $\Delta\Delta I$ are Positions 476, 482, and 485.

## Section C.1: Estimating the bias and variance of a relative entropy estimate

To take into account the impact of number of gaps on the amount of bias in the Relative Entropy value, we calculated the variance of the estimations arising from sample-size bias. The basic technique in [[11](#_ENREF_11)] is a delta approximation [[12](#_ENREF_12)]. Let a vector random variable $\mathbf{X}=\left(X_{1},X_{2},...,X_{s}\right)$ have expectations and covariances . Truncation of the multivariable Taylor expansion yields

$f\left(\mathbf{X}\right)\approx f\left(\mathbf{\mu}\right)+\sum_{j=1}^{s} \frac{\partial f\left(\mathbf{\mu}\right)}{\partial\mu_{j}}\left(X_{j}-\mu_{j}\right)+\frac{1}{2}\sum_{i=1}^{s} \sum_{j=1}^{s} \frac{\partial^{2}f\left(\mathbf{\mu}\right)}{\partial\mu_{i}\partial\mu_{j}}\left(X_{i}-\mu_{i}\right)\left(X_{j}-\mu_{j}\right)$.

In a normal approximation using the average of $n$ samples, the expectations of successive terms are typically of order $1,0,n^{-1},\mathrm{o}\left(n^{-1}\right),...$. Let $\hat{\mathbf{\mu}}$ be the sample mean. The “delta method” just expands to the second term in Eq and uses as an estimator. The estimator has approximate variance

.

To capture bias of $O\left(n^{-1}\right)$, Basharin [[11](#_ENREF_11)] takes the approximation to the third term in Eq , yielding

.

Define the Kronecker delta: $\delta_{i,j}=1$ if $i=j$ and 0 otherwise. To apply Eqs and to the relative entropy, consider two empirical probability distributions on an alphabet of $s$ letters, $\left\{\hat{p}_{j}=m_{j}/m:1\leq j\leq s\right\}$ and $\left\{\hat{q}_{j}=n_{j}/n:1\leq j\leq s\right\}$, where $m=\sum m_{j}$ and $n=\sum n_{j}$; $m_{j}$ ($n_{j}$) is an empirical count for the $j$-th letter. Then, standard results on the multinomial distribution give, the true probability of the $j$-th letter, and$\mathrm{cov}\left(\hat{p}_{i},\hat{p}_{j}\right)=\hat{p}_{i}\left(\delta_{i,j}-\hat{p}_{j}\right)/m$; and similarly for $\left\{\hat{q}_{j}\right\}$ and $n$. Note: $\mathrm{cov}\left(\hat{p}_{i},\hat{q}_{j}\right)=0$ if $\left\{\hat{p}_{j}\right\}$ and $\left\{\hat{q}_{j}\right\}$ are independent estimates of the corresponding distributions.

The relative entropy of $\left\{q_{j}\right\}$ with respect to $\left\{p_{j}\right\}$ is $\hat{I}=\sum q_{i}\ln\left(q_{i}/p_{i}\right)$. For the empirical relative entropy $\hat{I}=\sum\hat{q}_{i}\ln\left(\hat{q}_{i}/\hat{p}_{i}\right)$, the refined delta approximation in Eq gives

,

where $\sum q_{i}=\sum p_{i}=1$ gives the final equality. Note: the algebra of $\left\{\hat{q}_{j}\right\}$ accords with Basharin’s results for the entropy. Thus,

$$\hat{I}-\frac{s-1}{2}m^{-1}-\frac{1}{2}\left(-1+\sum\hat{q}_{i}\hat{p}_{i}^{-1}\right)n^{-1}$$

has approximate expectation 0, making it an (approximately) unbiased estimator of $I$. In the context of the RNA Nef problem, the object would be to check the size of $m^{-1}$ and $n^{-1}$ to ensure that the bias in using the sample estimate is small.

If so, the estimator in Eq has a variance dominated in practice by the variance of $\hat{I}$. Denote $H=-\sum q_{i}\ln q_{i}$. According to Eq , the variance in the delta method is

$\begin{matrix} \mathrm{var}\hat{I}\approx\sum\left(1+\ln q_{i}\right)^{2}\frac{q_{i}}{n}-\sum\sum\left(1+\ln q_{i}\right)\left(1+\ln q_{j}\right)\frac{q_{i}q_{j}}{n} \\ \text{ }-\sum q_{i}^{2}p_{i}^{-2}\frac{p_{i}\left(1-p_{i}\right)}{m}-\sum\ln^{2}p_{i}\frac{q_{i}\left(1-q_{i}\right)}{n} \\ =n^{-1}\left\{\sum\left(1+\ln q_{i}\right)^{2}q_{i}-\left[\sum\left(1+\ln q_{i}\right)q_{i}\right]^{2}-\sum q_{i}\left(1-q_{i}\right)\ln^{2}p_{i}\right\} \\ \text{ }-m^{-1}\sum q_{i}^{2}p_{i}^{-1}\left(1-p_{i}\right) \\ =n^{-1}\left\{1-2H+\sum q_{i}\ln^{2}q_{i}-\left(1-H\right)^{2}\right\}-m^{-1}\sum q_{i}^{2}\left(p_{i}^{-1}-1\right) \\ =n^{-1}\left\{\sum q_{i}\ln^{2}q_{i}-\left(\sum q_{i}\ln q_{i}\right)^{2}-\sum q_{i}\left(1-q_{i}\right)\ln^{2}p_{i}\right\}-m^{-1}\sum q_{i}^{2}\left(p_{i}^{-1}-1\right) \end{matrix}$.

# Section D: Globally and locally thermodynamically stable RNA G-quadruplexes within region R1

ViennaRNA predicted both locally stable (100-nt long segments) and globally stable (the complete 621-nt long Nef RNA sequence) G4s in the Nef RNA sequences we examined (See S3 Table for details). Our results suggested that the location of both locally and globally stable G4s is highly conserved both within and between the populations of Nef RNA sequences, mostly concentrated in two locations: 27-39 and 186-200. In the first location (27-39), the frequency of G4 did not differ much from Historic to Modern sequences (21/335 to 18/335, two-tailed Fisher Exact p = 0.74, 6% to 5%). In the second location (186-200), the frequency dropped significantly (from 187/335 to 89/335, p = 1.6x10^‒14^, 56% to 27%). The column corresponding to this location is marked in blue in S3 Table. Both locally and globally optimal G4s occurred in the same locations despite different windowing positions. To avoid redundancy in representation, S3 Table includes only subsequences with the positions most relevant to the G4s. Other surrounding sequences segments, not shown here, generally indicated the same G4. Unlike a much smaller change observed in location (27-39), the local test corresponding to the second location (186-200) confirmed a significant decrease in stable G4s in the Modern Nef RNAs (257/335 to 137/335, p = 3.0x10^‒21^, 76.7% to 40.9%).

1. Steffen P, Voss B, Rehmsmeier M, Reeder J, Giegerich R: **RNAshapes: an integrated RNA analysis package based on abstract shapes**. *Bioinformatics (Oxford, England)* 2006, **22**(4):500-503.

2. Quarta G, Kim N, Izzo JA, Schlick T: **Analysis of riboswitch structure and function by an energy landscape framework**. *Journal of molecular biology* 2009, **393**(4):993-1003.

3. Quarta G, Sin K, Schlick T: **Dynamic energy landscapes of riboswitches help interpret conformational rearrangements and function**. *PLoS computational biology* 2012, **8**(2):e1002368.

4. Kirchhoff F, Schindler M, Bailer N, Renkema GH, Saksela K, Knoop V, Müller-Trutwin MC, Santiago ML, Bibollet-Ruche F, Dittmar MT *et al*: **Nef Proteins from Simian Immunodeficiency Virus-Infected Chimpanzees Interact with p21-Activated Kinase 2 and Modulate Cell Surface Expression of Various Human Receptors**. *J Virol* 2004, **78**(13):6864-6874.

5. Coordinators NR: **Database resources of the National Center for Biotechnology Information**. *Nucleic acids research* 2013, **41**(Database issue):D8-D20.

6. Benson DA, Karsch-Mizrachi I, Lipman DJ, Ostell J, Sayers EW: **GenBank**. *Nucleic acids research* 2009, **37**(Database issue):D26-31.

7. Madden T: **The BLAST Sequence Analysis Tool**. In*.*, 2nd edn. Bethesda (MD): National Center for Biotechnology Information (US): The NCBI Handbook [Internet]; 2013-.

8. Griffiths-Jones S, Moxon S, Marshall M, Khanna A, Eddy SR, Bateman A: **Rfam: annotating non-coding RNAs in complete genomes**. *Nucleic acids research* 2005, **33**(Database issue):D121-124.

9. Peleg O, Trifonov EN, Bolshoy A: **Hidden messages in the nef gene of human immunodeficiency virus type 1 suggest a novel RNA secondary structure**. *Nucleic acids research* 2003, **31**(14):4192-4200.

10. Rice P, Longden I, Bleasby A: **EMBOSS: the European Molecular Biology Open Software Suite**. *Trends in genetics : TIG* 2000, **16**(6):276-277.

11. Basharin GP: **On a statistical estimate for the entropy of a sequence of independent random variables**. *Theory Prob Appl* 1959, **4**(3):333-336.

12. Papanicolaou A: **Taylor Approximation and the Delta Method**. In*.*: Stanford University; 2009.
